# Quantifying antimicrobial access and usage for paediatric diarrhoeal disease in an urban community setting in Asia

**DOI:** 10.1093/jac/dky231

**Published:** 2018-07-04

**Authors:** Le Thi Quynh Nhi, Ruklanthi de Alwis, Phung Khanh Lam, Nguyen Nhon Hoa, Nguyen Minh Nhan, Le Thi Tu Oanh, Dang Thanh Nam, Bui Nguyen Ngoc Han, Hoang Thi Thuy Huyen, Dinh Thi Tuyen, Vu Thuy Duong, Lu Lan Vi, Bui Thi Thuy Tien, Hoang Thi Diem Tuyet, Le Hoang Nha, Guy E Thwaites, Do Van Dung, Stephen Baker

**Affiliations:** 1 The Hospital for Tropical Diseases, Wellcome Trust Major Overseas Programme, Oxford University Clinical Research Unit, Ho Chi Minh City, Vietnam; 2 University of Medicine and Pharmacy in Ho Chi Minh City, Ho Chi Minh City, Vietnam; 3 Centre for Tropical Medicine, Nuffield Department of Clinical Medicine, Oxford University, Oxford, UK; 4 Children Hospital 1, Ho Chi Minh City, Vietnam; 5 The Hospital for Tropical Diseases, Ho Chi Minh City, Vietnam; 6 Hung Vuong Hospital, Ho Chi Minh City, Vietnam; 7 Ho Chi Minh City Department of Health, Ho Chi Minh City, Vietnam; 8 The Department of Medicine, University of Cambridge, Cambridge, UK

## Abstract

**Objectives:**

Antimicrobial-resistant infections are a major global health issue. Ease of antimicrobial access in developing countries is proposed to be a key driver of the antimicrobial resistance (AMR) epidemic despite a lack of community antimicrobial usage data.

**Methods:**

Using a mixed-methods approach (geospatial mapping, simulated clients, healthcare utilization, longitudinal cohort) we assessed antimicrobial access in the community and quantified antimicrobial usage for childhood diarrhoea in an urban Vietnamese setting.

**Results:**

The study area had a pharmacy density of 15.7 pharmacies/km^2^ (a pharmacy for every 1316 people). Using a simulated client method at pharmacies within the area, we found that 8% (3/37) and 22% (8/37) of outlets sold antimicrobials for paediatric watery and mucoid diarrhoea, respectively. However, despite ease of pharmacy access, the majority of caregivers would choose to take their child to a healthcare facility, with 81% (319/396) and 88% (347/396) of responders selecting a specialized hospital as one of their top three preferences when seeking treatment for watery and mucoid diarrhoea, respectively. We calculated that at least 19% (2688/14427) of diarrhoea episodes in those aged 1 to <5 years would receive an antimicrobial annually; however, antimicrobial usage was almost 10 times greater in hospitals than in the community.

**Conclusions:**

Our data question the impact of community antimicrobial usage on AMR and highlight the need for better education and guidelines for all professionals with the authority to prescribe antimicrobials.

## Introduction

Antimicrobial resistance (AMR) is rapidly becoming a global public health issue.^1^ In the past decade, many of the key antimicrobials on which we have come to rely in human medicine are in the process of losing, or have already lost, their effectiveness in treating infections caused by bacterial pathogens.^2^^,^^3^ AMR is a complex scientific, political, economic and social issue, and although developed countries are leading research efforts to tackle the problems, the greatest impact of AMR is currently being felt in low- and middle-income countries (LMICs).^4–6^ AMR is magnified in LMICs as antimicrobial access and usage, poor sanitation and limited access to clean water are amongst the principal drivers. Therefore, unrestricted access to low-cost antimicrobials combined with a high burden of infectious disease, limited diagnostic capacity and large urbanizing populations demanding better medical care has created a favourable environment for propagating antimicrobial-resistant bacteria in LMICs.^7^^,^^8^

Antimicrobials are frequently used indiscriminately to treat common human diseases, of which diarrhoea is a major contributor. It has been estimated that the worldwide burden of diarrhoea was >1.7 billion episodes in 2010, with the vast majority of these episodes arising in children in LMICs.^9^ The aetiological agents of diarrhoea are broad and can induce an array of symptoms and severity, including watery, mucoid and bloody diarrhoea. Currently, the WHO recommends oral rehydration solution (ORS) and zinc for all diarrhoea, and antimicrobial treatment for those with more severe symptoms.^10^^,^^11^ A large disease burden, combined with a lack of financial and diagnostic resources in LMICs, means that the causative agent is rarely or never identified.^12^ In Asia, this lack of a confirmative diagnosis results in the empirical use of antimicrobials in the community or a healthcare facility. However, many of those receiving antimicrobials for diarrhoea may not require them,^13^ as the disease is generally self-limiting and frequently of viral aetiology,^12^ and AMR in diarrhoeagenic bacteria is now highly prevalent in LMICs in Asia.^3^^,^^13^

Despite the perceived haphazard use of antimicrobials in LMICs, antimicrobial access and usage for the treatment of common infections in vulnerable populations has seldom been accurately estimated. Such data are vital for understanding how AMR may emerge, spread and be sustained, and are critical for targeting public health interventions to reduce antimicrobial usage. Vietnam is a rapidly developing LMIC in Southeast Asia with a population of >90 million people.^14^ An increasing population, a high infectious disease burden and a largely unregulated antimicrobial market combine to make Vietnam the ideal location for investigating antimicrobial access and usage. Diarrhoeal disease is a common childhood illness in Vietnam, a country with high recorded antimicrobial use in the healthcare system.^13^^,^^15^ By combining geospatial analysis, community and pharmacy surveys, and a longitudinal cohort study, we aimed to investigate antimicrobial access and usage for paediatric diarrhoeal disease in the community and the healthcare system in an urban setting in Vietnam.

## Materials and methods

### Ethics

Ethics approvals were provided by the Oxford University Tropical Research Ethics Committee (OxTREC approval 5110-16) and the institutional review board of the Ho Chi Minh City (HCMC) University of Medicine and Pharmacy (No. 220/DHYD-HD). Written informed consent was obtained from all participants (from parents and guardians if the participant was <18 years old).

### Study setting and design

With an estimated population of >8 million people, HCMC is the largest city in Vietnam.^16^ HCMC is divided into five rural districts and 19 urban districts that cover an area of 19 km^2^; the urban districts had a mean population density of 13394 people/km^2^ in 2016.^16^ We selected District 8 (hereinafter referred to as the study area), with 16 wards and a population density of 22522 people/km^2^, as the location for sampling owing to its urban setting and central location (Figure S1, available as Supplementary data at *JAC* Online, and Table 1).^16^ To assess antimicrobial access and usage we triangulated data from a prospective paediatric cohort^17^ and conducted a cross-sectional study with several components: (i) geographical mapping of all pharmacies and healthcare facilities in the study area; (ii) a pharmacy practice survey to assess medication suggested by retail pharmacies for paediatric diarrhoea; and (iii) a self-reported caregiver community survey on the management and antimicrobial treatment of paediatric diarrhoea.

**Table 1.** Pharmacies and healthcare clinics in the various wards in the study area

			Population^a^		Pharmacy density (per 10000 people)	
Ward in District 8	Healthcare facilities	Pharmacies	all	1 to <5 years	all	1 to <5 years
1	7	10	25782	944	3.88	105.93
2	20	29	20694	843	14.01	344.01
3	15	16	24803	1139	6.45	140.47
4	51	36	42200	2284	8.53	157.62
5	46	48	40177	2538	11.95	189.13
6	15	34	30144	1353	11.28	251.29
7	12	28	31685	1414	8.84	198.02
8	4	5	9024	444	5.54	112.61
9	8	14	21564	1194	6.49	117.25
10	15	14	15841	836	8.84	167.46
11	7	5	7591	294	6.59	170.07
12	7	10	16653	816	6.00	122.55
13	12	7	8809	480	7.95	145.83
14	4	6	20575	1144	2.92	52.45
15	10	13	39623	2354	3.28	55.23
16	6	26	41010	1243	6.34	209.17
Total	239	301	396175	19320	7.59	142

a Data from HCMC Demographic Department, 30 June 2017.

The coordinates of all pharmacies in the study area were recorded and entered into EpiCollect5.^18^ Vietnam administrative boundaries were downloaded from Global Administrative Divisions Map (https://gadm.org/download_country_v3.html). Vietnam population data rasters (population per 0.01 km^2^) were downloaded from WorldPop (http://www.worldpop.org.uk/data/WorldPop_data/AllContinents/130_metadata.html; 100 m resolution), and satellite imagery of Vietnam was downloaded from ESRI (http://www.esrivn.com/en/index.html) basemaps. All geospatial mapping was conducted using ArcGIS version 10.2 (Redlands, CA, USA). Euclidean distance to the nearest pharmacy and kernel density plots for pharmacy density (i.e. pharmacies per 0.01 km^2^) were estimated for the study area using the ‘Euclidean distance’ and ‘Kernel’ ArcGIS tools, respectively.

### Pharmacy practice survey

We conducted a survey of pharmacy behaviour using an unbiased observational technique for assessing dispensed medication known as ‘simulated client methodology’.^19–22^ Two ‘mystery shoppers’ with no healthcare training role-played as caregivers and approached pharmacies in a sub-region (Ward 5) of the study area to seek medical advice and treatment for two childhood diarrhoea scenarios. Scenario 1 (watery diarrhoea): a mother attending the pharmacies in the study area to buy medication for a child aged 2 years. The child has had 4–5 loose stool episodes within the previous 24 h. The child has no fever, and no blood or mucus in stools. Scenario 2 (mucoid diarrhoea with fever): a mother attending the pharmacies in the study area to buy medication for a child aged 2 years. The child has had 4–5 loose stool episodes within the previous 24 h. The child had a mild fever and mucus in the stool. Pharmacy-prescribed drugs were labelled anonymously, and then were classified by a clinician and confirmed by a qualified pharmacist (scripts in Table S1). In addition we aimed to investigate whether antimicrobials were being sold without a doctor’s prescription by sending a mystery shopper to pharmacies in the study area to buy ciprofloxacin. This was a specific scenario in which the shopper requested this antimicrobial and did not provide any information about for what or whom the treatment was required.

### Self-reported behaviour survey

We performed a community survey to capture the self-reported behaviour of parents and caregivers in managing diarrhoea in their children aged between 1 and 5 years. The surveyed population consisted of participants residing in the study area. We randomly sampled a representative population stratified by 16 wards and with registered children aged <5 years. Local health authorities accompanied and assisted in community visits. Mothers or caregivers were asked a short questionnaire regarding their choice of care if their child had diarrhoea in simulated scenarios similar to those described in the pharmacy behaviour study. We surveyed 396 parents or caregivers (Table S2). Results from these questionnaires are reported as means (interquartile range) and frequencies (%) for continuous and categorical variables respectively. For comparisons between groups, we used Fisher’s exact test for categorical variables, and the Mann–Whitney *U*-test for continuous variables (significant *P* ≤ 0.05).

**Table 2.** The demographic characteristics of parents and caregivers who chose a pharmacy or a hospital as first choice for a child with diarrhoea

	Watery diarrhoea			Mucoid diarrhoea		
	pharmacy (*n *=* *87)	hospital (*n *=* *89)	*P* value^a^	pharmacy (*n *=* *65)	hospital (*n *=* *142)	*P* value^a^
Female sex	73/87 (84)	72/89 (81)	0.693	55/65 (85)	113/142 (80)	0.448
Role of parent (not caregivers)	54/86 (63)	39/89 (44)	**0.015**	43/64 (67)	69/142 (49)	**0.015**
Age in years, mean (IQR)	38 (30–50)^b^	40 (32–57)	0.051	38 (31–50)^c^	40 (32–56)^d^	0.198
Healthcare trained	7/83 (8)	11/83 (13)	0.461	4/63 (6)	16/138 (12)	0.316
Employment	87/87 (100)	89/89 (100)	0.661	65/65 (100)	142/142 (100)	0.943
worker	10/87 (11)	10/89 (11)		6/65 (9)	16/142 (11)	
officer	6/87 (7)	6/89 (7)		5/65 (8)	10/142 (7)	
health staff	2/87 (2)	6/89 (7)		1/65 (2)	6/142 (4)	
business owner	15/87 (17)	10/89 (11)		11/65 (17)	23/142 (16)	
housewife	35/87 (40)	34/89 (38)		29/65 (45)	56/142 (39)	
other (retired)	19/87 (22)	23/89 (26)		13/65 (20)	31/142 (22)	
Education ≤12 years	75/87 (86)	68/88 (77)	0.171	55/65 (85)	115/141 (82)	0.695
Monthly income (VND)^e^			0.406			0.739
≤1.3 million	15/78 (19)	14/80 (18)		11/57 (19)	20/127 (16)	
1.3–9 million	59/78 (76)	57/80 (71)		42/57 (74)	95/127 (75)	
>9 million	4/78 (5)	9/80 (11)		4/57 (7)	12/127 (9)	
Only 1 child	36/84 (43)	37/84 (44)	1.000	21/65 (32)	61/134 (46)	0.091
Only 1 child aged <5 years	68/85 (80)	71/86 (83)	0.699	49/65 (75)	105/134 (78)	0.718
Age of first child, years, mean (IQR)	3 (2–4)	2/87 (2–4)^f^	0.486	3 (2–4)	3/140 (2–4)^g^	0.553
Infections in previous month	57/86 (66)	44/86 (51)	0.063	43/65 (66)	73/139 (53)	0.071
Medication in previous month	60/87 (69)	51/87 (59)	0.207	45/65 (69)	83/141 (59)	0.167
Antimicrobials in previous month	24/60 (40)	26/70 (37)	0.857	18/41 (44)	46/118 (39)	0.585

All values shown are *n*/*N* (%) unless otherwise indicated.

a *P* values calculated using Fisher’s exact test for categorical variables and the Mann–Whitney *U*-test for continuous variables. Significant *P* values are highlighted in bold.

b Number of respondents = 81.

c Number of respondents = 60.

d Number of respondents = 139.

e Monthly income <1300000 VND/month is determined to be poor and below average (as suggested by the Vietnamese government), whereas income ≥9000000 VND/month is considered a good monthly income and above average.

f Number of respondents = 87.

g Number of respondents = 140.

### Quantifying antimicrobial use for diarrhoea

**Figure 1.**
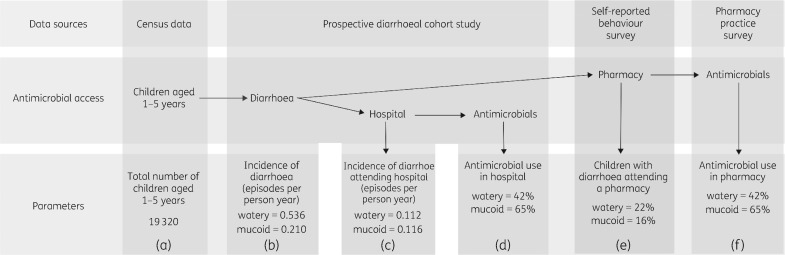
Estimating antimicrobial usage for diarrhoea in children aged 1–5 years in pharmacies and hospital. Diagram outlining the sources of data, parameters, and calculations used to quantify antimicrobial usage for diarrhoea in children aged 1–5 years in this population. Note each of the parameters have been given an initial (A–F), which informs the calculations in Table 3.

We considered that antimicrobial usage for diarrhoeal disease was dependent on the number of diarrhoeal episodes for which medical treatment was sought, and how likely antimicrobials were to be prescribed for diarrhoeal treatment. Figure 1 outlines the parameters, the source of data and the calculations used to quantify antimicrobial usage for diarrhoea in children aged 1–5 years. The annual number of diarrhoeal episodes presenting at pharmacies was the product of (A) the total number of children aged 1–5 years in the study area, (B) the incidence rate of diarrhoea episodes per child-year, and (E) the proportion of parents or caregivers selecting to first take their child to a pharmacy. The annual number of diarrhoeal episodes presenting at hospitals was multiplied by (A) the total number of children aged 1–5 years living in the study area and (C) the incidence rate of diarrhoea episodes presenting at hospital per child-year. Parameter (A) was derived from recent census data from the HCMC Demographic department (30 June 2017). Incidence rates (B) and (C) were estimated from a prospective diarrhoeal cohort conducted between 2014 and 2016 that actively followed up 748 children residing in the study area.^17^ The proportion of parents or caregivers seeking pharmacies as their first choice (E) was derived from the self-reported behaviour survey of parents and caregivers. To quantify the likelihood of prescribing antimicrobials for diarrhoeal treatment at a hospital or a pharmacy, we used antimicrobial treatment data from (D) the prospective cohort study^17^ (from hospital files) and (F) the pharmacy behaviour study, respectively.

## Results

### The distribution of outlets selling antimicrobials

**Figure 2.**
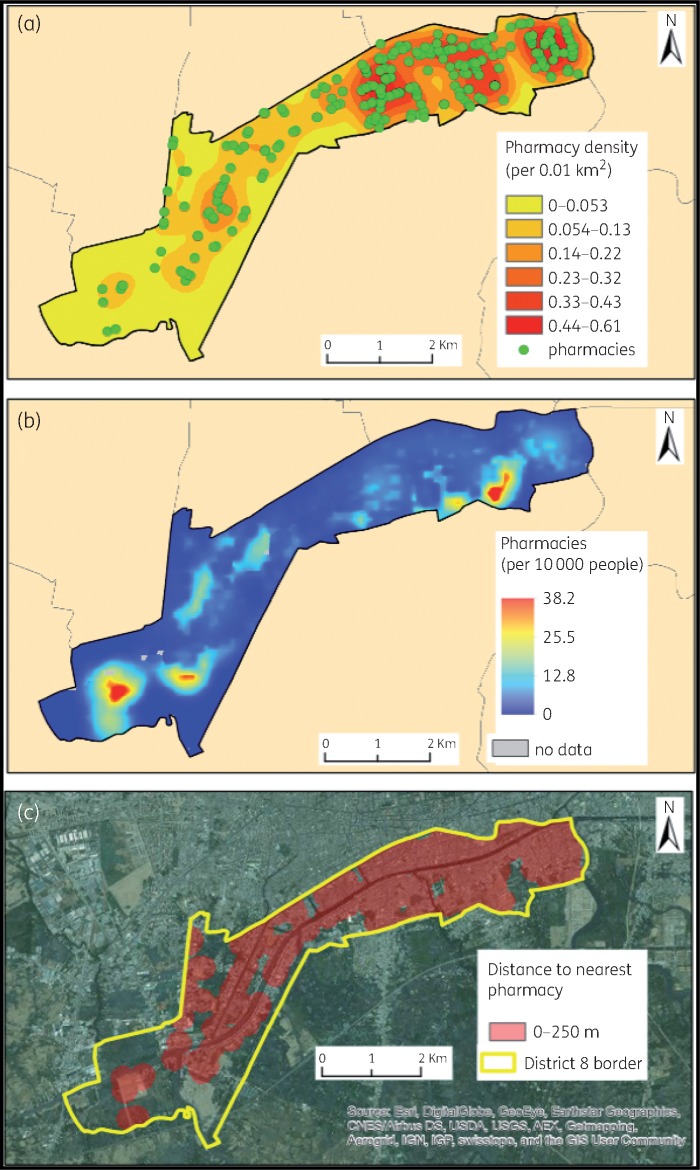
The geographical distribution of pharmacies in the Ho Chi Minh City study area. Maps of district 8 in Ho Chi Minh City. (a) The point locations of pharmacies and the kernel distribution of pharmacy shop density. Green points identify the pharmacy shops and pharmacy density is determined by colour intensity (see key). (b) The distribution of pharmacy shops density per 10 000 inhabitants. The greater the intensity of red the higher the number of pharmacy shops per 10 000 people (see key, areas in grey are those with no data owing to missing data in the population raster). (c) The geographical areas in District 8 that are within 250 m of a pharmacy. Red shaded areas are locations in which residents live with 250 m of a location selling antimicrobials.

We mapped a total of 301 pharmacies and 243 private or public healthcare clinics in the study area (Table 1 and Figure 2a). Figure S1 shows the geographical location of the study area. The distribution of pharmacies was not uniform and ranged from 0 to 61 pharmacies/km^2^, with a mean density of 15.7 pharmacies/km^2^. The majority of outlets were located on main thoroughfares in the north of the study area (Figure 2a). The population pharmacy density equated to 7.59 pharmacies/10000 people, corresponding to a pharmacy for every 1316 people. The number of pharmacies within the population varied from 0 to 38.2 pharmacies/10000 people, with four high-density areas (>30 outlets/10000 people) (Figure 2b). The high density of pharmacy shops epitomized routine access to antimicrobials in this community and equated to >75% of the study area population living within 250 m of an outlet selling antimicrobials (Figure 2c).

### Pharmacy prescribing practices for diarrhoea

**Figure 3.**
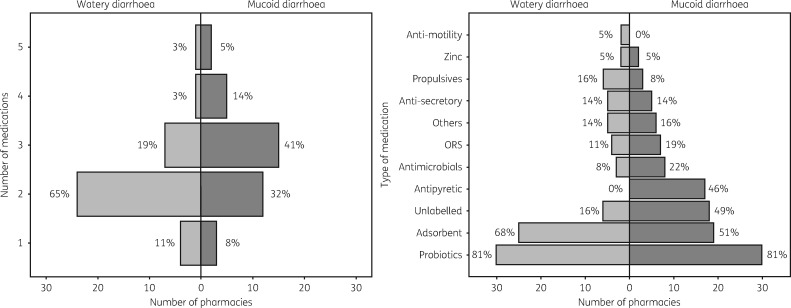
Medications sold by pharmacies in the study area for paediatric diarrhoea. (a) Rotated histogram showing the number and proportion (*x*-axis) of the 37 pharmacies visited during the ‘mystery shopper’ simulated client method selling between 1 and 5 medications (*y*-axis) for watery and mucoid diarrhoea, respectively. (b) Rotated histogram showing the number and proportion (*x*-axis) of the 37 pharmacies visited during the ‘mystery shopper’ simulated client method selling different mediations (*y*-axis) for watery and mucoid diarrhoea, respectively.

We surveyed 48 pharmacy shops (37 were open when visited) in a ward within the study area (Figure S1), and used two different scenarios for paediatric diarrhoea. In the first scenario (watery diarrhoea), the number of prescribed medications ranged from one to five, with the majority (65%; 24/37) of pharmacies prescribing two different medications (Figure 3a). The median total cost for each pharmacy visit was 17000 Vietnamese dong (VND) (US $1 ≈ 23000 VND); range 7000–54000 VND. A total of 46 different types and brands of medications were sold, which could be broadly categorized into 11 classes: anti-motility, zinc, anti-emetics, anti-secretory, probiotics, ORS, antimicrobial, antipyretic, adsorbent, unlabelled and others. Unlabelled treatments were prescribed in 16% (6/37) of pharmacy visits, with probiotics (81%; 30/37) and adsorbents (68%; 25/37) being the most commonly sold treatments. Antimicrobials were prescribed by 3/37 (8%) pharmacies for watery diarrhoea (Figure 3b).

In the second scenario (mucoid diarrhoea with fever), 53 different medications were sold by the 37 pharmacies; undefined/unlabelled medications were sold in 49% (18/37) of pharmacy visits (Figure 3b). Probiotics (81%; 30/37) and adsorbents (51%; 19/37) remained the most common medications prescribed, but the proportion of pharmacies prescribing an antimicrobial increased to 22% (8/37). There was also an increase in the number of different mediations sold for mucoid diarrhoea in comparison with those sold to treat watery diarrhoea (Figure 3a), with a higher proportion of pharmacies selling a combination of three (41%; 15/37), four (14%; 5/37) or five (5%; 2/37) medications. Despite there being a greater number of medications sold, the cost to caregivers in this scenario was not substantially greater than with watery diarrhoea (median 18000 VND; range 3000–31000 VND).

There was considerable disparity in the type of antimicrobials sold in the 37 surveyed pharmacies, with pharmacies prescribing trimethoprim sulphate, sulphonamides, fluoroquinolones, macrolides and cephalosporins. When the mystery shopper returned to the same pharmacies a week later and asked to buy ciprofloxacin, 100% (37/37) of the vendors sold the requested antimicrobial. This medication was consistently sold without dosing advice and without the caregiver having to provide information as to why the antimicrobial was required. Generic ciprofloxacin was sold in a range of different packaging from 14 different manufacturers in two different concentrations.

## Antimicrobial usage surveys in caregivers

To evaluate how local residents in the study area access antimicrobials and to understand their knowledge, attitudes and practices regarding antimicrobial usage, we surveyed 396 individuals (Table S2). The majority (82%; 325/396) of the interviewees were women and the median age was 39 years (range 19–87 years). Ten percent of participants (39/381) indicated some background in healthcare. The majority (69%; 243/351) had an average household income (≥1300000 and ≤9000000 VND/household/month), with 21% (74/351) reporting being in a poor financial situation (<1300000 VND/household/month). Most caregivers/parents (60%; 232/386) had ≥2 children living in the household. Many (59%; 231/394) reported that their children had received medication for a medical condition within the last month, of which approximately half (47%; 109/231) were reported to be an antimicrobial.

We found that a majority of interviewed parents and caregivers had limited knowledge about antimicrobials. Many assumed that antimicrobials could be generically used to treat coughing (46%; 181/396), fever (35%; 137/396), colds (30%; 118/396), headaches (21%; 81/396) and diarrhoea (19%; 76/396). Approximately half (51%; 203/396) of the parents and caregivers agreed with the statement ‘some infections can be difficult to treat if there is antimicrobial resistance’, and 59% (233/396) agreed with the statement ‘resistance can occur if you do not take a sufficient dose’. In self-reported prescribing, more than half of parents and caregivers (54%; 213/394) said they had at some time purchased antimicrobials from pharmacies, with many of them (85%; 182/213) buying antimicrobials within the 30 days prior to the interview (Table S3).

**Table 3.** The burden of diarrhoea and antimicrobial treatment for diarrhoea in children aged 1–5 years

	Diarrhoea episodes per year (95% CI)				
Parameter	total	attending hospital	receiving an antimicrobial in hospital	attending a pharmacy	receiving an antimicrobial at a pharmacy
Calculation^a^	(A × B)	(A × C)	(A × C × D)	(A × B × E)	(A × B × E × F)
Diarrhoea type					
watery	10355 (8945–11765)	2163 (1622–2724)	898 (673–1130)	2275 (1965–2584)	184 (159–209)
mucoid	4057 (2975–5158)	2241 (1603–2878)	1462 (1046–1878)	667 (489–848)	144 (105–183)
Total	14427 (12636–16 219)	4404 (3560–5249)	2359 (1884–2834)	2945 (2585–3305)	329 (282–375)

a Calculation derived from parameters described in Figure 1.

## Diarrhoeal treatment-seeking behaviour

**Figure 4.**
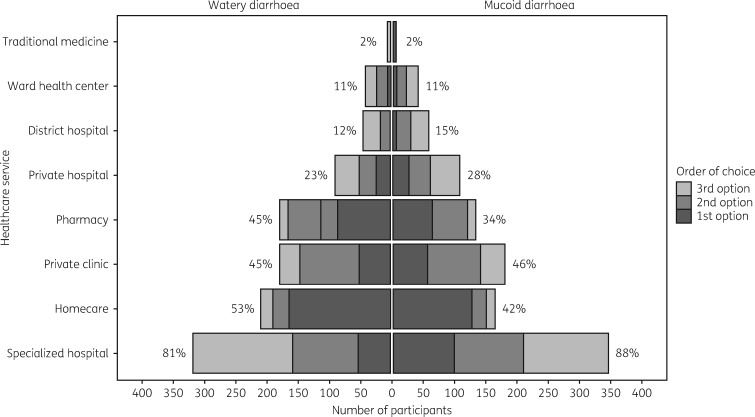
Healthcare utilization for paediatric diarrhoea by caregivers. Pyramid plot showing the number and proportions of 396 individual caregivers or parents of children aged <5 years residing in the study area (*x*-axis) selecting the various healthcare services (*y*-axis) as first, second or third choices (see key for shading) for watery and mucoid diarrhoea.

Figure 4 shows the top three preferences for places where parents and caregivers would seek healthcare advice if their children had either of the two previously described diarrhoeal disease scenarios. In both scenarios, the most common first choice was to treat their children at home. However, a specialized hospital was the most common option, with 81% (319/396) and 88% (347/396) of responders selecting this as one of their top three preferences for treatment of watery and mucoid diarrhoea, respectively. Visiting a local pharmacy as a first choice ranked third for watery diarrhoea and second for mucoid diarrhoea. Overall, visiting a pharmacy was selected as one of the top three preferences by 45% (180/396) and 34% (134/396) of caregivers for watery and mucoid diarrhoea, respectively (Figure 4). We observed that parents (rather than a caregivers) and those of younger age were more likely to take their children to a pharmacy than a hospital for watery diarrhoea (*P = *0.015 and *P = *0.051, respectively). Similarly, parents (rather than caregivers) were significantly more likely to take their children to a pharmacy than a hospital for mucoid diarrhoea (Table 2).

## Quantifying antimicrobial use for diarrhoea

We lastly aimed to calculate antimicrobial usage in the community and in hospitals for diarrhoeal disease in children aged between 1 and 5 years (Figure 1). From following 748 children and quantifying episodes of diarrhoea over a 2 year period in the diarrhoea cohort, we estimated the total annual incidence of watery and mucoid diarrhoea to be 536 (95% CI 463–609) and 210 (95% CI 154–267) per 1000 child-years, respectively (Table 3). The annual incidences of watery and mucoid diarrhoea resulting in hospital attendance were 112 (95% CI 83–141) and 116 (95% CI 83–149) per 1000 child-years, respectively. Among the diarrhoeal episodes resulting in hospital attendance, 66/159 (42%) watery episodes and 107/164 (65%) mucoid episodes received antimicrobial treatment in hospital. We therefore estimated the total number of diarrhoeal episodes within the study area in this age group to be 10355 (95% CI 8945–11765) and 4057 (95% CI 2975–5158) per year for watery and mucoid diarrhoea, respectively.

Combining data from the surveys of the self-reported behaviour and the pharmacy practice, we estimated that the annual number of diarrhoeal episodes in children aged 1–5 years that would receive antimicrobial treatment at pharmacies were 184 (95% CI 159–209) for watery diarrhoea and 144 (95% CI 105–183) for mucoid diarrhoea (Table 3). However, data from the diarrhoea cohort indicated that the number of paediatric diarrhoeal cases that resulted in an antimicrobial administration in a hospital was approximately 5 [898 (95% CI 673–1130)] and 10 times higher [1462 (95% CI 1046–1878)] for watery and mucoid diarrhoea, respectively (Table 3). In total, we estimated that at least 19% (2688/14 427) of diarrhoeal episodes in children aged 1–5 years in the study area would receive an antimicrobial annually.

## Discussion

AMR infections are increasingly common throughout Asia, a trend that may be driven in part by access to antimicrobials.^23–25^ Vietnam illustrates this trend; it is the world’s 15th most populous country, urbanization has increased from 28% in 2014 to 34.9% in 2017 and it is predicted to have a population of >100 million people by 2025.^26^ Antimicrobial dispensing in Vietnam is known to be poorly regulated, leading to inappropriate prescribing and self-medication for a number of common infections, including fever and acute respiratory infections.^27^^,^^28^ We selected childhood diarrhoea, which is common in Vietnam, and among the top motives for seeking healthcare in HCMC, as a syndrome for which to assess antimicrobial use.^12^^,^^29–31^

The overall density of pharmacies in the community in HCMC is high (7.59/10000 people), and outnumbers the number of pharmacy staff that have been trained at any level (assistant or university degree) in the city (5.98/10000 people).^32^ This density of pharmacies in HCMC is reflective of the general pattern in LMICs, for example Sabde *et al.*^33^ reported a comparable ratio in an urban area of India (5.84/10000 people). However, the number of pharmacy outlets in HCMC is more than double the mean density of pharmacies in other areas of the world, such as Europe (3.06/10000 people)^34^ and other parts of Southeast Asia (3.02/10000 people),^34^ and three times higher than that of some countries in the Western Pacific (2.28/10000 people)^34^ and the USA (2.11/10000 people).^35^ This ratio of pharmacies is six times higher than in LMICs in Africa.^34^ Ease of access to retail pharmacies may improve equity in the use of medication but it allows access to treatments that should be better controlled, especially when pharmacy staff are underqualified and/or fail to follow appropriate guidelines.

Our study demonstrated the common sale of antimicrobials without prescription for paediatric diarrhoeal disease in the community in HCMC. Notably, we estimated that antimicrobial sales for paediatric diarrhoeal disease in the community in HCMC were lower than in several other LMICs, such as Ethiopia (26%; 58/223),^20^ and other Asian countries, such as Pakistan (14%)^36^ and Thailand (52%).^37^ The low proportion of antimicrobials dispensed for acute watery diarrhoea in HCMC is corresponds with a declining trend in antimicrobials being used for watery diarrhoea in Vietnam. Limited available data suggest that the proportion of antimicrobials dispensed for acute watery disease has decreased over the last 20 years, from 45% of cases in 1997,^38^ to 14% in 2013,^39^ to 8% (3/37) here. This trend may result from a substantial improvement in practices in pharmacies to alleviative antimicrobial prescribing and the impact of resistance. However, despite an array of public health engagement and intervention projects, antimicrobials still remain the most inappropriate drugs dispensed from retail pharmacies in Vietnam.^40^ It is additionally worth noting that previous studies have suggested that antimicrobial usage is greater in rural areas than in urban areas.^28^

We predicted that the diarrhoeal disease management in the community in HCMC would be comparable with that of other LMICs, where antimicrobials are dispensed without further consideration.^36–38^^,^^41^^,^^42^ However, we found that the total number of diarrhoeal episodes that would receive an antimicrobial in a hospital was 5–10 times higher than in retail pharmacies. Our findings are consistent with a recent finding in this setting, where 85% of paediatric diarrhoeal patients admitted to hospital were prescribed an antimicrobial.^13^ It has been estimated that one-third of antimicrobial prescriptions in Vietnamese hospitals may be inappropriate,^43^ a level that could promote AMR in various bacterial pathogens. It is apparent that in a setting with limited-to-no diagnostic testing, this clinical approach is considered optimal for preventing complications and saving lives. There is a large demand for hospital care in HCMC, and as doctors have limited time to make clinical decisions, such an approach is deemed effective and less time consuming. Therefore, there is a clear requirement for tests that can identify children at the more severe end of the disease spectrum and can distinguish between a viral and a bacterial infection.

Our study has some limitations. We likely underestimated the overall antimicrobial use as our calculation did not take into account antimicrobial use in facilities other than specialized hospitals. Additionally, relying on the choice of attending pharmacy as the first option only and the practice of selling antimicrobials in a small number of pharmacies from two scenarios may underestimate antimicrobial sales in pharmacies. Even though we employed a thorough process to quantify antimicrobial use for diarrhoea, our data extrapolation may contain errors as we compiled data from several sources. In addition, as we aimed to assess antimicrobial dispensing only, we did not record or evaluate the retailer’s consulting abilities for using these treatments, which may reflect local antimicrobial handling practices. Lastly, our combined studies focused on diarrhoeal diseases in young children only, and we recognize that our approach offers limited understanding of antimicrobial usage in other populations and other presentations.

We conclude that the density of pharmacy retailers is extremely high in urban HCMC. Antimicrobial usage for diarrhoeal disease in children is more common in hospitals than via community pharmacies. Given these findings, combined with the general low level of knowledge regarding antimicrobials among parents and caregivers, we predict a high burden of antimicrobial overuse in Vietnam. We advocate better education, training and guidelines for antimicrobial usage and AMR for all health professionals prescribing antimicrobials in Vietnam.

## Supplementary Material

Supplementary Data
