# Supplementary material for: Quantifying antimicrobial access and usage for paediatric diarrhoeal disease in an urban community setting in Asia
Source: J Antimicrob Chemother. 2018 Jul 4;73(9):2546–54. doi: 10.1093/jac/dky231 (PMC6105870; doi:10.1093/jac/dky231)
Supplement: Supplementary Data [file dky231_supplementary_data.docx]

**Supplementary data**

| **Scenario** | **What** | **Who** | **How** |
| --- | --- | --- | --- |
| Scenario 1 | Buying medication for 2-year old child having watery diarrheal disease | Mother (M)  Drug vendor (V) | M: Greeting and asking for buying medication for my son.  V: May ask for age and symptoms of the son.  M: Response with these following information:   - 2 years old or 27 – 28 months - 4-5 loose stool since the last 24 hour   V: May ask for other symptoms such as blood or mucus in stool.  M: Response with “No blood or mucus seen”.  In case V doesn’t ask for this information, M doesn’t answer it.  V: May ask for other symptoms such as vomit and fever.  M: Response with these following information:   - No vomiting - No fever   V: May ask for how many days of medication the mother would want.  M: Response with “Any is fine.”  Closing the conversation by saying good bye and leaving. |
| Scenario 2 | Buying medication for 2-year old child having mucoid diarrheal disease | Mother (M)  Drug vendor (V) | M: Greeting and asking for buying medication for my son  V: May ask for age and symptoms of the son  M: Response with these following information:   - 2 years old or 27 – 28 months - 4-5 loose stool since the last 24 hour   V: May ask for other symptoms such as blood or mucus in stool.  M: Response with “Some mucus seen”  In case V doesn’t ask for this information, M added this information later in the conversation.  V: May ask for other symptoms such as vomit, fever.  M: Response with these following information:   - No vomiting - Mild fever   V: May ask for how mild the fever is.  M: Response with “ Warmer than usual”  V: May ask for how many days of medication the mother would want.  M: Response with “Any is fine.”  Closing the conversation by saying good bye and leaving. |

**Table S1**. Script for the mystery shopping in the pharmacy survey.

**Table S2.** The demographic characteristics of parents and caregivers in the community behaviour survey.

| **Characteristics** | **Number of responders** | **%** |
| --- | --- | --- |
| **Total** | 396 | 100 |
| **Female sex** | 325/396 | 82.1 |
| **Role** |  |  |
| Parents | 215/394 | 54.6 |
| Caregiver | 179/394 | 45.4 |
| **Age** **(years)** | 39 (31 – 54) |  |
| <41 | 209/384 | 54.4 |
| 41 – 60 | 131/384 | 34.1 |
| >60 | 44/384 | 11.5 |
| **Healthcare trained** | 39/381 | 10.2 |
| **Employment** |  |  |
| Worker | 50/396 | 12.6 |
| Officer | 35/396 | 8.8 |
| Health staff | 12/396 | 3.0 |
| Business owner | 70/396 | 17.7 |
| Housewife | 157/396 | 39.6 |
| Others (retired) | 76/396 | 19.2 |
| **Education (years)** |  |  |
| ≤ 12 | 304/393 | 77.2 |
| >12 | 89/393 | 22.6 |
| **Monthly income ^a^** |  |  |
| Below average | 74/381 | 19.4 |
| Average | 243/381 | 63.8 |
| Above average | 34/381 | 8.9 |
| **Number of children** |  |  |
| 1 | 154/386 | 39.9 |
| ≥ 2 | 232/386 | 60.1 |
| **Children <5 years** |  |  |
| 1 | 300/386 | 77.7 |
| ≥ 2 | 86/386 | 22.3 |
| **Age of child (years)** | 3 (2– 4) |  |
| **Infections in previous month** | 218/396 | 55.1 |
| **Medication last month** | 231/396 | 58.3 |
| **Current treatment** |  |  |
| Respiratory | 136/396 | 34.3 |
| Fever | 41/396 | 10.4 |
| Flu | 13/396 | 3.3 |
| Diarrhea | 5/396 | 1.3 |
| Others | 9/396 | 2.3 |
| **Antimicrobials in previous month** | 109/305 | 35.7 |

1. Monthly income <1,300,000VND/month is determined to be poor and below average (as suggested by the Vietnamese government), while >=9,000,000VND/month is a good monthly income and above average.

**Table S3.** Knowledge, attitudes, and practice of parents and caregivers toward antimicrobials and antimicrobial resistance.

| **Feature** | **Variable** | **Positive response** | **Proportion (%)** |
| --- | --- | --- | --- |

| **Knowledge** | Perceptions of antimicrobials treatment | Can be used for treating headaches | 81/396 | 20.5 |
| --- | --- | --- | --- | --- |
|  |  | Can be used for treating diarrhea | 76/396 | 19.2 |
|  |  | Can be used for treating teething | 36/396 | 9.1 |
|  |  | Can be used for treating fever | 137/396 | 34.6 |
|  |  | Can be used for treating colds | 118/396 | 29.8 |
|  |  | Can be used for treating coughing | 181/396 | 45.7 |
|  | Knowledge on prescription antimicrobials | Knowing that antimicrobials could be bought from doctors’ prescription | 305/396 | 77.0 |
|  |  | Knowing that antimicrobials could be got on request to doctor | 17/396 | 4.3 |
|  |  | Knowing that antimicrobials could be got on request to drug seller | 218/396 | 55.1 |
|  |  | Do not know how to have antimicrobials | 22/396 | 5.6 |
| **Attitude** | Some infections can be difficult to treat if there is antimicrobial resistance | Agree | 203/396 | 51.3 |
|  |  | Disagree | 23/396 | 5.8 |
|  |  | Do not know | 167/396 | 42.1 |
|  |  | Other | 3/396 | 0.8 |
|  | Resistance can happen if not taking enough doses | Agree | 233/396 | 58.8 |
|  |  | Disagree | 21/396 | 5.3 |
|  |  | Do not know | 139/396 | 35.1 |
|  |  | Other | 3/396 | 0.8 |
| **Practice** | Practice of buying drugs from pharmacies | Ever bought drugs from pharmacies | 357/395 | 90.4 |
|  |  | Ever bought antimicrobials from pharmacies | 213/394 | 54.1 |
|  | Time of buying antimicrobials | Ever bought antimicrobials during the last 30 days | 182/305 | 59.7 |
|  |  | Ever bought antimicrobials more than 6 months | 74/305 | 24.3 |
|  |  | Don’t remember the latest time of buying antimicrobials | 49/305 | 16.1 |


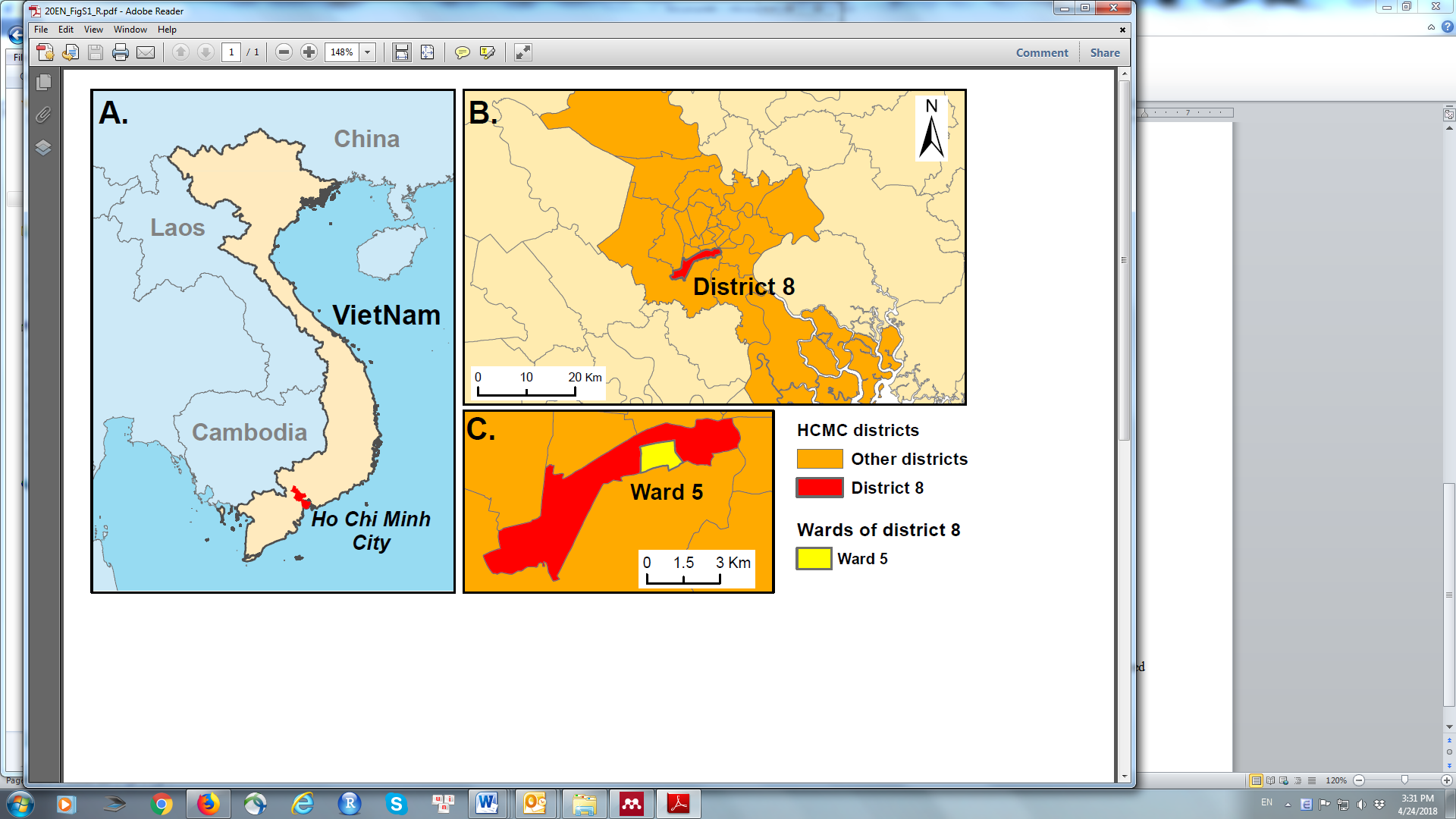


**Figure S1**. The geographical location for this study.

A) Map of Vietnam showing the location of the greater Ho Chi Minh City area. B) Enhanced map of Ho Chi Minh City showing the districts C) Enhanced map of district 8 (the study area) of Ho Chi Minh City highlighting ward 5 as the location for the “mystery shopper” simulated client’s method in yellow.

**Informed consent forms**

1. **ICFs for the diarrhea cohort**
   1. **English version for the potential participants coming from the birth cohort extension**

| 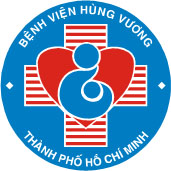 | **Hospital for Tropical Diseases**  Oxford University Clinical Research Unit  764 Vo Van Kiet, Quan 5, Ho Chi Minh City  **Viet Nam** | 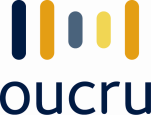 |
| --- | --- | --- |

Dr Nguyen Trong Hieu

0903159285

**Information sheet for the “Diarrhoeal Cohort Study”**

**For children who are enrolled in the original birth cohort extension**

**OXTREC – 1058-13**

**Information about this program**

We are inviting mothers and their children who have been enrolled in our “Healthy Children” program at Hung Vuong Hospital to be involved in **“Diarrhoeal Cohort Study”**. This form will give you the information you will need to help you decide whether or not to participate. Please read the form carefully. You may ask questions about this program, the possible risks and benefits, your rights as a volunteer, and anything related or this form that is not clear. When all of your questions have been answered, you can decide if you and your child will participate. This process is called ‘informed’ consent.

The aims of this research program are to identify how often young Vietnamese children get sick with diarrhoea, what causes the illness and why children get sick. Additionally, we are interested in understanding how your child’s blood responds to diarrhoeal diseases. We hope to learn more about diarrhoeal disease in Ho Chi Minh City so that we can make a vaccine to prevent disease in children in the future.

This program will be part of the current program you are already enrolled in. The specific goal of this new part will be to study a bacterium called *Shigella*, which often causes severe diarrhoea in young children. To learn more about *Shigella* and other diarrhoeal diseases in young Vietnamese children, we would like to collect information on where and how children live through a series of surveys. We will do this by following the health of your child for two years. During this time we will monitor your child’s blood for evidence of infection, and will ask you about any illness that your child and your family members have had, including any admission to hospital. The information we get from these surveys will help us to make a vaccine to prevent diarrhoeal disease due to *Shigella* in the future.

Part of this program will mean we will collect your child’s blood to perform genetic tests. These tests will help us to know why some people get sick with this disease, while others do not. If you agree, the genetic code in your child’s blood will be tested. The results of these tests will be made available to other researchers but you and your child will not be identified. Some samples will be sent out of the country for testing, which may be considered to be an invasion of privacy. Providing blood for these tests is optional. If you do not agree for these genetic tests to be conducted, you (or your child) may still take part in the study.

**Who is doing this program ?**

The partners in the study are Hung Vuong Hospital and Hospital for Tropical Diseases.

If I consent on behalf of my child, what will happen to my child and me in this program and what are the risks?

**At enrolment (today):**

When you attend the HVH-WBC for your routine follow up appointment as part of the Healthy Baby Cohort program in early 2014, our nurse will invite you to participate in the diarrhoeal birth cohort. If you agree for you and your child to participate in this new program our nurse will review previously collected information about where you live, your family structure, and the child’s health status. For the existing “Healthy Children” program, the nurse will collect a 2ml blood sample from your child. A maximum of 2ml of blood will be collected from your child today. The nurse will also collect a stool sample, or an anal swab if stool is unavailable, from your child. Finally, the nurse will collect a respiratory swab from your child’s nose.

**At eachfollow-up visit (every 6 months for 2 years):**

In order to monitor your child’s health and development, we need to see you and your child one time every six months. Your child will be aged between 18-36 months at the time of enrolment, meaning this study will finish when your child is aged between 42-60 months.

Each visit will take approximately half an hour, and will take place here at the same clinic where you are enrolled. The nurse will give you a card to remind you of when you should visit for follow-up visits. At each follow-up visit the baby will receive a health check from a hospital doctor. We will record some simple information about your child’s development and also ask you some simple questions about your child’s health. When your child is 18-35 months of age, we would like to collect 2ml of blood (about 20 drops) from your child. When your child is 3660 months of age, we would like to collect 3 ml of blood (about 30 drops) from your child. Only one blood sample will be drawn from your child at each visit. A highly experienced nurse will collect the blood using a small needle from your child’s hand. This will hurt for a moment, and might leave a small bruise. At each follow-up visit the nurse will also collect a stool sample or anal swab and a respiratory swab from your child. These procedures are quick and painless.

**When you are home with your child and you think your child is sick with diarrhoea;**

We will send you a short SMS message routinely to ask if your child has experienced an episode of diarrhoea. If you respond with “yes”, ournurses will call you to arrange a convenient time to visit your house to collect a stool sample, ask a set of simple questions and provide medical advice to help your child. If you feel that you need to seek hospitalization, we ask that you consider bringing your child to the Hospital for Tropical Diseases. If you attend the Hospital for Tropical Diseases when your child has diarrhoea, we request that you call our nurse so she can support you and your child when he/she is evaluated by a doctor from HTD. You can also bring your child to other clinics or hospitals that are convenient for you.

**Risks and benefits to being in the program:**

By being in the program, you will have access to experienced doctors in the Well Baby Clinic at Hung Vuong hospital who will examine your child for no cost at scheduled visits that will monitor your child’s health and development. The amount of blood we collect from your infant at each visit is small (20-30 drops). Your child will probably cry out when the sample is collected. You will receive money to compensate you for travel costs in bringing your child to the Well Babyclinic, HVH for follow-up visits in addition to free diagnostic services and supported travel costs for diarrhoeal examinations at HTD. If you bring your child to a private clinic or an emergency department of the hospital other than HTD when your child is sick with diarrhoea, compensation will not be given.

**What we will do with the blood and other samples we collect from your child**

The blood and stool samples we collect from your child will be used for research. The specimens will be stored in a freezer. We will do various tests on the blood, stool and respiratory samples we collect from your child to understand if your child has ever had *Shigella* or other diarrhoeal and respiratory diseases that are common in Vietnam. The tests we do on the blood and respiratory samples we collect from your child will not have a direct benefit to you, but might help other children in the future. Samples we collect from your child may be used in future studies.

**Confidentiality**

All of the information we get from you is strictly private. Your child’s name will not be on any samples we collect or test results–we will use a number instead of a name. Your name, or your child’s name, will not be mentioned in any output from this program. We will ask for information on the district and ward in which you live, but we will not use this information for any purpose outside this program or give it to anyone else.

**Costs**

There will be no cost to you for participating in this program. The costs of examination by a paediatrician at scheduled program visits will be paid for by the program. You will be compensated for the travel costs you incur by bringing your child to the clinic for scheduled program visits.

**Voluntary participation**

If you do not want to be part of this program, it will not affect the care your child will receive in any way. If you do agree to become a program participant, you can withdraw from the program at any time (verbally). The care your child receives will not be affected. However, information collected on your child up until you withdraw will still be used. If you choose not to participate in this program, your child will not be monitored for diarrhoeal illness. However, you will still be able to participate in the current Healthy Baby Cohort Extension that your child is already enrolled in.

**End of the program**

We will stop seeing your child at HVHafter two years of follow up. No information on your child health and no samples will be collected after this time.

**Obtaining additional information**

- You are encouraged to ask any questions related to this program during the time of participation. If you have any questions about this program, its procedures, risks and benefits, or alternatives please call Dr. Nguyễn Trọng Hiếu at 0903 159 285.
- If you have any questions about your child’s rights as a subject in this program, you may want to talk to Dr. Lu Lan Vi, or if you want to speak to someone outside of the program you may contact the Ethics Committee at the HTD at 083 855 8532.

**Consent form for the “Diarrhoeal Cohort Study’ OXTREC -1058-13**

**Consent from: MOTHER/FATHER/GUARDIAN**

- I have been fully informed of the possible risks and benefits of taking part in this diarrhoeal cohort programand agree that I and my child will take part.
- I know who to contact if I need more information. I understand that confidentiality will be preserved. I understand that I am free to withdraw from the program at any time without affecting the care I or my child will receive.
- I understand there will be limited direct benefit to me or my child.
- I agree to allow nurses to come to my home when I report a diarrhoeal disease episode in my child
- I agree that the geographical location of my house may be recorded in order to understand any relationship between location and disease.

**□ I AGREE OR □ I DO NOT AGREE** that the samples taken can be stored for other research studies about infectious diseases, including genetic testing and these tests may be done outside of Viet Nam.

Participant Number: BCB- ___ - ___ ___ ___ ___

**Participant’s name: ____________________________________________**

**_____________________________________________ _____________________________________________**

**Signature of person giving consent Relationship to participant**

**______________________________________________ ____________________________________________
Print name Date of signature**

**Investigator’s statement**

I, the undersigned, have fully explained the relevant information of this research to the participant named above and will provide her/him with a copy of this signed and dated informed consent form.

____________________________________ ____________________________________ _________________________

**Investigator / designee signature Print name Date of signature**

**If the person giving consent cannot read the form, a witness must be present and sign here:**

I was present throughout the entire informed consent process with the participant. All questions from the participant were answered and the participant has agreed to take part in the research.

____________________________________ ____________________________________ _________________________

**Witness signature Print name Date of signature**

**Consent for genetic testing OXTREC – 1058-13**

**(to be signed by the participant’s parent or guardian)**

By signing/marking my name here, I confirm my willingness for my child’s blood to be used for genetic tests.

Participant Number: BCB- ___ - ___ ___ ___ ___

**Participant’s name: ____________________________________________**

**_____________________________________________ _____________________________________________**

**Signature of person giving consent Relationship to participant**

**______________________________________________ ____________________________________________
Print name Date of signature**

I, the undersigned, have fully explained the relevant information of this research to the participant named above and will provide her/him with a copy of this signed and dated informed consent form.

____________________________________ ____________________________________ _________________________

Investigator / designee signature Print name Date of signature

**If the person giving consent cannot read the form her/himself, a witness must be present and sign here:**

I was present throughout the entire informed consent process with the participant. All questions from the participant were answered and the participant has agreed to take part in the research.

____________________________________ ____________________________________ _________________________

Witness signature Print name Date of signature

- 1. **Vietnamese version for the potential participants coming from the birth cohort extension**

| 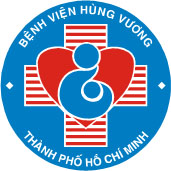 | **Bệnh viện Bệnh Nhiệt Đới**  Đơn vị Nghiên cứu Lâm sàng Đại học Oxford  764 Võ Văn Kiệt, Quận 5, Thành phố Hồ Chí Minh  **Việt Nam** | 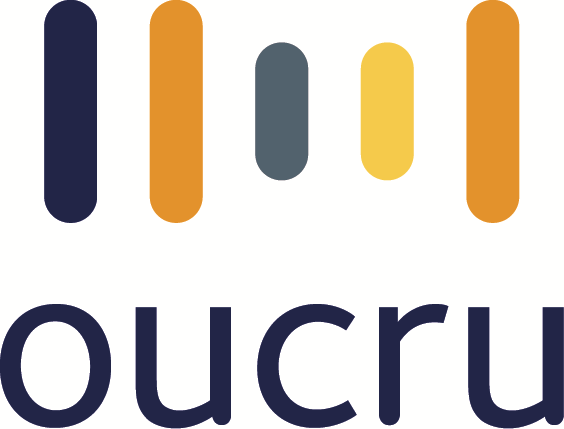 |
| --- | --- | --- |

Bs. Nguyễn Trọng Hiếu

0903159285

**Phiếu thông tin cho “Nghiên cứu Đoàn hệ Bệnh Tiêu chảy”**

**Cho trẻ đã tham gia vào nghiên cứu mở rộng của "Sức khỏe trẻ em"**

**OXTREC-1058-13**

**Thông tin về chương trình**

Chúng tôi mời những bà mẹ và em bé đã tham gia trong chương trình "Sức Khỏe Trẻ Em" của chúng tôi tại Bệnh viện Hùng Vương cùng tham gia vào **"Nghiên Cứu Đoàn Hệ Bệnh Tiêu Chảy"**. Phiếu này sẽ cung cấp cho bạn những thông tin cần thiết để bạn quyết định có tham gia hay không. Vui lòng đọc kỹ tờ thông tin này. Bạn có thể hỏi về chương trình này, những lợi ích và nguy cơ có thể xảy ra, quyền của người tham gia nghiên cứu, và bất kỳ vấn đề nào có liên quan hay thông tin trên phiếu này mà bạn thấy chưa rõ. Khi mọi thắc mắc đã được giải đáp, bạn có thể quyết định cho bạn và con bạn có tham gia hay không. Quá trình này được gọi là sự lấy chấp thuận tham gia nghiên cứu.

Mục tiêu của chương trình này là để xác định tần suất bị bệnh tiêu chảy ở trẻ em Việt Nam, nguyên nhân gây tiêu chảy và tại sao trẻ em lại bị tiêu chảy. Ngoài ra chúng tôi cũng muốn tìm hiểu về việc máu của con bạn phản ứng với bệnh tiêu chảy như thế nào. Chúng tôi hy vọng có thể hiểu hơn về bệnh tiêu chảy ở thành phố Hồ Chí Minh để có thể tạo vắc xin giúp các trẻ em ngừa bệnh sau này.

Đây là một phần của chương trình “Sức khỏe trẻ em”mà bạn đang tham gia. Mục tiêu cụ thể của phần mới này là để tìm hiểu một chủng vi khuẩn có tên gọi *Shigella*, thường gây bệnh tiêu chảy nặng ở trẻ nhỏ. Chúng tôi cần khảo sát thu thập thông tin về nơi ở và điều kiện sống của trẻ em để hiểu hơn về bệnh do vi khuẩn Shigella và các nguyên nhân gây bệnh tiêu chảy khác ở trẻ em Việt Nam. Để thực hiện được điều này, chúng tôi sẽ theo dõi sức khỏe của con bạn trong 2 năm. Trong suốt thời gian đó chúng tôi sẽ theo dõi máu của bé để tìm chứng cứ của nhiễm khuẩn, và sẽ hỏi bạn về tất cả những bệnh mà bé và các thành viên gia đình bạn mắc phải, kể cả những lần phải nhập viện. Những thông tin có được từ các khảo sátnày có thể giúp chúng tôi trong việc tạo ra vắc xin ngăn ngừa bệnh tiêu chảy do nhiễm khuẩn *Shigella* trong tương lai.

Chương trình này cũng sẽ có một phần thực hiện các xét nghiệm di truyền trên mẫu máu của con bạn. Xét nghiệm di truyền này giúp chúng ta hiểu vì sao một số người bị bệnh này trong khi một số khác thì không bị. Nếu bạn đồng ý, thông tin di truyền trong máu của con bạn sẽ được xét nghiệm. Kết quả xét nghiệm này sẽ được sử dụng để tiến hành các nghiên cứu khác, tuy nhiên danh tính của bạn và con bạn sẽ được ẩn để không ai biết thông tin di truyền đó là của ai. Một số mẫu sẽ được gửi ra nước ngoài để xét nghiệm, có thể điều này được cho là xâm phạm đến sự riêng tư. Việc đồng ý cho máu để xét nghiệm di truyền là tùy ở bạn. Bạn và con bạn vẫn có thể tham gia vào chương trình này dù bạn không đồng ý để mẫu máu được dùng cho xét nghiệm di truyền.

**Các đơn vị tham gia chương trình ?**

Các đơn vị cộng tác tham gia nghiên cứu bao gồm Bệnh viện Hùng Vương (TPHCM) và Bệnh viện Bệnh Nhiệt Đới (TPHCM).

Nếu như tôi thay mặt con tôi chấp thuận tham gia, điều gì sẽ xảy ra trong chương trình và có những nguy cơ thế nào?

**Khi tham gia (hôm nay):**

Khi bạn đưa bé đến hẹn khám theo dõi cho nghiên cứu Sức Khỏe Trẻ Em ở Phòng khám nhi - Bệnh viện Hùng Vương vào đầu năm 2014, bạn và con bạn sẽ được mời tham gia vào "Nghiên cứu Đoàn hệ Bệnh Tiêu Chảy". Nếu bạn và con bạn đồng ý tham gia, một điều dưỡng sẽ cập nhập những thông tin về bạn như nơi bạn ở, cấu trúc gia đình, và tình trạng sức khỏe của bé. Điều dưỡng cũng sẽ lấy 2ml mẫu máu của bé cho chương trình "Sức khỏe trẻ em". Chỉ lấy tối đa 2ml máu từ bé trong lần khám này. Điều dưỡng cũng sẽ lấy một mẫu phân, hoặc một mẫu phết hậu môn nếu không có phân, và mẫu phết mũi họng của con bạn.

**Các lần tái khám (mỗi 6 tháng một lần trong 2 năm):**

Để theo dõi tình trạng sức khỏe và sự phát triển của con bạn, chúng tôi cần gặp bạn và bé vào mỗi 6 tháng một lần. Quá trình theo dõi như sau: nếu con bạn tham gia chương trình này lúc 18 tháng tuổi thì lần khám theo dõi cuối cùng là khi bé được 42 tháng tuổi; nếu tham gia lúc 24 tháng tuổi thì lần khám cuối là khi 48 tháng tuổi; nếu tham gia lúc 30 tháng tuổi thì lần khám cuối là khi 54 tháng tuổi; nếu tham gia lúc 36 tháng tuổi thì lần khám cuối là khi 60 tháng tuổi.

Mỗi lần tái khám mất khoảng nửa tiếng tại ngay phòng khám nơi bạn được nhận vào nghiên cứu. Điều dưỡng sẽ phát cho bạn một thẻ ghi lịch tái khám của chương trình. Tại mỗi lần tái khám, con của bạn sẽ được bác sĩ kiểm tra sức khỏe. Chúng tôi sẽ ghi lại những thông tin về sự phát triển của con bạn và hỏi thêm một số câu hỏi đơn giản về sức khỏe của bé. Khi con bạn được 18 – 35 tháng tuổi, chúng tôi sẽ lấy khoảng 2ml máu (khoảng 20 giọt) của con bạn cho nghiên cứu. Khi con bạn được 36 – 60 tháng tuổi chúng tôi sẽ lấy 3ml máu (khoảng 30 giọt). Mỗi lần khám chỉ lấy 1 mẫu máu của bé. Một điều dưỡng nhiều kinh nghiệm sẽ dùng cây kim nhỏ để lấy máu trên tay của bé. Việc lấy máu sẽ gây đau một tí và có thể lại một vết bầm nhỏ. Tại mỗi lần tái khám, điều dưỡng cũng sẽ lấy một mẫu phân hoặc phết hậu môn và một mẫu phết mũi họng của bé. Việc thu mẫu này rất nhanh và không gây đau.

**Khi bạn ở nhà với bé và bạn nghĩ rằng bé bị bệnh tiêu chảy;**

Chúng tôi sẽ giữ liên lạc với bạn bằng tin nhắn SMS định kỳ để hỏi thăm bé có bị tiêu chảy không. Nếu bạn trả lời "Có", một điều dưỡng của chúng tôi sẽ gọi điện cho bạn để sắp xếp thời gian thuận tiện để đến thăm nhà bạn để lấy một mẫu phân, hỏi một số câu hỏi đơn giản và tư vấn y tế để giúp con bạn. Nếu bạn cảm thấy rằng con bạn cần nhập viện, chúng tôi mong bạn xem xét đưa bé đến Bệnh viện Bệnh Nhiệt Đới. Nếu bạn muốn đưa bé đến Bệnh viện Bệnh Nhiệt Đới khi bé bị tiêu chảy, hãy gọi cho điều dưỡng của chúng tôi để được hỗ trợ và bé sẽ được khám bởi một bác sĩ chuyên khoa củabệnh viện Bệnh Nhiệt đới. Chúng tôi sẽ thu thập một mẫu phân của bé khi bạn cho bé đến khám tại đây. Bạn cũng có thể đưa bé đến các phòng khám hoặc bệnh viện khác nếu điều đó thuận tiện cho bạn.

**Các nguy cơ và lợi ích khi tham gia:**

Khi tham gia vào chương trình, con bạn sẽ được những bác sĩ có kinh nghiệm tại phòng khám Nhi bệnh viện Hùng Vương khám và tư vấn miễn phí vào những lần tái khám định kỳ để theo dõi tình trạng sức khỏe và sự phát triển của bé. Lượng máu chúng tôi lấy từ con bạn tại mỗi lần tái khám là rất nhỏ (khoảng 20-30 giọt). Bé có thể khóc khi bị lấy máu. Bạn sẽ nhận được một khoản tiền hỗ trợ cho chi phí đi lại cho các lần tái khám tại phòng khám Nhi, Bệnh viện Hùng Vương, các lần đi khám bệnh vì tiêu chảy tại bệnh viện bệnh Nhiệt đới cũng như miễn phí xét nghiệm khi đến khám tại bệnh viện này vì tiêu chảy. Nếu bạn cho con đến khám ở một phòng khám tư hoặc đến khoa cấp cứu tại một bệnh viện khác, không phải là Bệnh viện Bệnh Nhiệt đới khi bé bị tiêu chảy, chúng tôi sẽ không hỗ trợ các chi phí này.

**Chúng tôi sẽ làm gì với máu và các mẫu khác thu từ con bạn**

Mẫu máu và phân mà chúng tôi thu được từ con của bạn sẽ được dùng vào nghiên cứu. Các mẫu đó sẽ được lưu trữ trong tủ đông lạnh. Chúng tôi sẽ làm một số xét nghiệm trên mẫu máu, phân và mẫu phết mũi họng của con bạn để xem con bạn có từng bị nhiễm *Shigella* hoặc một số bệnh tiêu chảy và hô hấp phổ biến khác ở Việt Nam hay không. Những xét nghiệm mà chúng tôi tiến hành trên các mẫu máu và mũi họng thu được từ con bạn có thể không mang lại lợi ích trực tiếp cho bạn, nhưng điều này có thể giúp những trẻ em khác trong tương lai. Các mẫu chúng tôi thu được từ con bạn cũng có thể được sử dụng cho các nghiên cứu về sau.

**Tính bảo mật**

Tất cả những thông tin chúng tôi có được từ bạn sẽ được bảo mật nghiêm ngặt. Tên của con bạn sẽ không hiện diện trên bất kì một mẫu xét nghiệm nào cũng như trên kết quả của chúng tôi - chúng tôi sẽ dùng mã số thay cho tên. Tên của bạn, tên của con bạn sẽ không được đề cập đến trên kết quả của chương trình . Chúng tôi sẽ hỏi thông tin về quận và phường nơi bạn ở nhưng sẽ không sử dụng thông tin này vào bất kỳ mục đích nào khác ngoài chương trình này và cũng không đưa cho ai khác.

**Chi phí**

Bạn không tốn bất kì chi phí nào khi tham gia vào chương trìnhnày. Việc khám bệnh theo lịch sẽ được chương trình chi trả. Bạn sẽ được hỗ trợ chi phí đi lại khi mang bé đến phòng khám để tái khám theo lịch đã hẹn. Bạn cũng được miễn phí chi phí xét nghiệm chẩn đoán bệnh và được hỗ trợ chi phí đi lại khi cho bé đến khám tại bệnh viện Bệnh Nhiệt Đới vì tiêu chảy.

**Sự tự nguyện tham gia chương trình**

Dù bạn chọn không tham gia vào chương trình thì việc đó cũng không ảnh hưởng đến việc chăm sóc sức khỏe cho con bạn. Ngay cả khi bạn đã đồng ý tham gia chương trình, bạn cũng có thể xin rút khỏi chương trình bất kỳ lúc nào (bằng lời). Việc chăm sóc sức khỏe cho con bạn sẽ không bị ảnh hưởng. Tuy nhiên những thông tin đã được thu thập về con bạn cho đến thời điểm đó vẫn sẽ được dùng cho nghiên cứu. Nếu bạn chọn không tham gia vào nghiên cứu này, con bạn sẽ không được theo dõi bệnh tiêu chảy. Tuy nhiên, con bạn vẫn có thể tiếp tục tham gia nghiên cứu Sức khỏe Trẻ em mở rộng.

**Kết thúc chương trình**

Chúng tôi sẽ ngừng khám theo dõi con bạn sau 2 năm. Sau đó, chúng tôi sẽ không thu thập thêm mẫu và thông tin từ con bạn nữa.

**Các thông tin thêm**

- Chúng tôi khuyến khích bạn hỏi thêm bất kỳ câu hỏi gì liên quan đến chương trình này trong suốt thời gian tham gia. Nếu bạn có thắc mắc về chương trình, các quy trình, nguy cơ và lợi ích, hay các câu hỏi khác, vui lòng gọi BS. Nguyễn Trọng Hiếu tại số 0903 159 285.
- Nếu bạn có bất kỳ thắc mắc gì về các quyền của con bạn khi tham gia chương trình này, bạn có thể liên hệ BS. Lư Lan Vi , hoặc nếu bạn muốn nói chuyện với ai khác ngoài nhóm nghiên cứu, bạn có thể liên hệ với Hội Đồng Đạo Đức tại Bệnh Nhiệt đới ở số 083 855 8532.

**Phiếu chấp thuận tham gia "Nghiên cứu Đoàn hệ Bệnh Tiêu chảy" OXTREC – 1058-13**

**Chấp thuận từ: BỐ/MẸ/ NGƯỜI GIÁM HỘ**

- Tôi đã được thông tin đầy đủ về các nguy cơ và lợi ích có thể có của việc cho con tôi tham gia vào chương trình nghiên cứu đoàn hệ bệnh tiêu chảy này và đồng ý rằng tôi và con của tôi sẽ tham gia.
- Tôi biết sẽ liên lạc với ai khi cần hỏi thêm thông tin. Tôi hiểu rằng các thông tin về tôi hay con tôi sẽ được bảo mật. Tôi hiểu được tôi có quyền rút khỏi chương trình vào bất kỳ lúc nào mà không ảnh hưởng đến việc chăm sóc sức khỏe mà tôi hay con tôi sẽ được nhận.
- Tôi hiểu là việc nghiên cứu này sẽ không mang đến nhiều lợi ích trực tiếp cho tôi và con tôi
- Tôi đồng ý để điều dưỡng của chương trình đến nhà tôi khi tôi báo con tôi bị nhiễm bệnh tiêu chảy
- Tôi đồng ý việc định vị nhà ở của tôi có thể được thực hiện để tìm hiểu sự liên quan giữa nơi ở và bệnh tật.

**□ Tôi đồng ý** hoặc **□** **tôi không đồng ý** cho phép mẫu của con tôi được lưu trữ cho các nghiên cứu về sau trên các bệnh truyền nhiễm, bao gồm các xét nghiệm di truyền và các xét nghiệm này có thể được thực hiện bên ngoài Việt Nam.

Mã số tham gia: 14EN - ___ - ___ ___ ___ ___

**Tên người tham gia: ____________________________________________**

**______________________________________ ______________________________________**

**Chữ ký của người chấp thuận Quan hệ với người tham gia**

**______________________________________ _____________________________________**

**Họ Tên (chữ in) Ngày ký**

**Xác nhận của nghiên cứu viên**

Tôi, người ký tên bên dưới, đã giải thích đầy đủ các thông tin liên quan đến chương trình nghiên cứu này cho người tham gia có tên bên trên và sẽ cung cấp cho cô/anh ấy một bản sao của phiếu chấp thuận đã được ký và ghi ngày tháng này.

**______________________________ _____________________________ ____________________________**

**Chữ ký của nghiên cứu viên Họ Tên (chữ in) Ngày ký**

**hoặc người được chỉ định**

**Nếu người cho chấp thuận không thể tự đọc phiếu này, một nhân chứng phải có mặt và ký tên dưới đây:**

Tôi đã có mặt cùng với người tham gia trong suốt quá trình lấy chấp thuận. Tất cả các câu hỏi của người tham gia đã được trả lời và người tham gia đã đồng ý tham gia vào chương trình nghiên cứu.

**______________________________ ______________________________**  **__________________________**

**Chữ ký nhân chứng Họ Tên (chữ in) Ngày ký**

**Phiếu chấp thuận cho xét nghiệm di truyền OXTREC - 1058-13**

**(được ký tên bởi bố mẹ hoặc người giám hộ của trẻ tham gia nghiên cứu)**

Bằng việc ký tên/đánh dấu ở đây, tôi xác nhận đồng ý cho phép mẫu máu của con tôi được dùng vào các xét nghiệm di truyền.

Mã số tham gia: 14EN-___-___ ___ ___ ___

**Tên người tham gia: ____________________________________________**

**______________________________________ ______________________________________**

**Chữ ký của người chấp thuận Quan hệ với người tham gia**

**______________________________________ ______________________________________**

**Họ Tên (chữ in) Ngày ký**

Tôi, người ký tên bên dưới, đã giải thích đầy đủ các thông tin liên quan đến nghiên cứu này cho người tham gia có tên bên trên và sẽ cung cấp cho cô/anh ấy một bản sao của phiếu chấp thuận đã được ký và ghi ngày tháng này.

**______________________________ _____________________________ ____________________________**

**Chữ ký của nghiên cứu viên Họ Tên (chữ in) Ngày ký**

**hoặc người được chỉ định**

**Nếu người cho chấp thuận không thể tự đọc phiếu này, một nhân chứng phải có mặt và ký tên dưới đây:**

Tôi đã có mặt cùng với người tham gia trong suốt quá trình lấy chấp thuận. Tất cả các câu hỏi của người tham gia đã được trả lời và người tham gia đã đồng ý tham gia vào chương trình nghiên cứu.

**______________________________ ______________________________**  **__________________________**

**Chữ ký nhân chứng Họ Tên (chữ in) Ngày ký**

- 1. **English version for the potential participants coming from the orginal cohort**

| 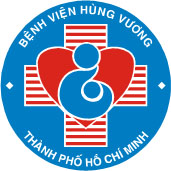 | **Hospital for Tropical Diseases**  Oxford University Clinical Research Unit  764 Vo Van Kiet, Quan 5, Ho Chi Minh City  **Viet Nam** | 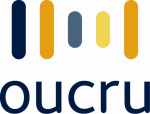 |
| --- | --- | --- |

Dr Nguyen Trong Hieu

0903159285

**Information sheet for the “Diarrhoeal Cohort Study”**

**For children who are enrolled in the original birth cohort**

**OXTREC – 1058-13**

**Information about this program**

We are inviting mothers and their children who have been enrolled in our “Healthy Children” program at Hung Vuong Hospital to be involved in **“Diarrhoeal Cohort Study”**. This form will give you the information you will need to help you decide whether or not to participate. Please read the form carefully. You may ask questions about this program, the possible risks and benefits, your rights as a volunteer, and anything related or this form that is not clear. When all of your questions have been answered, you can decide if you and your child will participate. This process is called ‘informed’ consent.

The aims of this program are to identify how often young Vietnamese children get sick with diarrhoea, what causes the illness and why children get sick. Additionally, we are interested in understanding how your child’s blood responds to diarrhoeal diseases. We hope to learn more about diarrhoeal disease in Ho Chi Minh City so that we can make a vaccine to prevent disease in children in the future.

This will be part of the current program you are already enrolled in. The specific goal of this new part of the program will be to study a bacterium called *Shigella*, which often causes severe diarrhoea in young children. To learn more about *Shigella* and other diarrhoeal diseases in young Vietnamese children, we would like to collect information on where and how children live through a series of surveys. We will do this by following the health of your child for two years. During this time we will monitor your child’s blood for evidence of infection, and will ask you about any illness that your child and your family members have had, including any admission to hospital. The information we get from these surveys will help us to make a vaccine to prevent diarrhoeal disease due to *Shigella* in the future.

Part of this program will mean we will collect your child’s blood to perform genetic tests. These tests will help us to know why some people get sick with this disease, while others do not. If you agree, the genetic code in your child’s blood will be tested. The results of these tests will be made available to other researchers but you and your child will not be identified. Some samples will be sent out of the country for testing, which may be considered to be an invasion of privacy. Providing blood for these tests is optional. If you do not agree for these genetic tests to be conducted, you (or your child) may still take part in the study.

**Who is doing this program?**

The partners in the study are Hung Vuong Hospital and Hospital for Tropical Diseases.

If I consent on behalf of my child, what will happen to my child and me in this program and what are the risks?

**At enrolment (today):**

When you attend the HVH-WBC for your final routine follow up appointment as part of the Healthy Baby Cohort program at 12 months of age, our nurse will invite you to participate in the diarrhoeal birth cohort. If you agree for you and your child to participate in this new program, a nurse will review previously collected information about where you live, your family structure, and the child’s health status. For the existing “Healthy Children” program, the nurse will collect a 2ml blood sample (about 20 drops) from your child, instead of the 1ml sample (about 10 drops) that would otherwise have been collected. A maximum of 2ml of blood will be collected from your child today. The nurse will also collect a stool sample, or an anal swab if stool is unavailable, from your child. Finally, the nurse will collect a respiratory swab from your child’s nose.

**At each follow-up visit (every 6 months for 2 years):**

In order to monitor your child’s health and development, we need to see you and your child one time every six months, at 18, 24, 30 and 36 months of age.

Each visit will take approximately half an hour, and will take place here at the same clinic where you are enrolled. The nurse will give you a card to remind you of when you should visit for follow-up visits. At each follow-up visit the baby will receive a health check from a hospital doctor. We will record some simple information about your child’s development and also ask you some simple questions about your child’s health. When your child is 18, 24, and 30 months of age, we would like to collect 2ml of blood (about 20 drops) from your child. When your child is 36 months of age, we would like to collect 3 ml of blood (about 30 drops) from your child. Only one blood sample will be drawn from your child during this visit. A highly experienced nurse will collect the blood using a small needle from your child’s hand. This will hurt for a moment, and might leave a small bruise. At each follow-up visit the nurse will also collect a stool sample or anal swab and a respiratory swab from your child. These procedures are quick and painless.

**When you are home with your child and you think your child is sick with diarrhoea;**

We will send you a short SMS message routinely to ask if your child has experienced an episode of diarrhoea. If you respond with “yes”, our nurses will call you to arrange a convenient time to visit your house to collect a stool sample, ask a set of simple questions and provide medical advice to help your child. If you feel that you need to seek hospitalization, we ask that you consider bringing your child to the Hospital for Tropical Diseases. If you attend the Hospital for Tropical Diseases when your child has diarrhoea, we request that you call our nurse so she can support you and your child when he/she is evaluated by a doctor from HTD. You can also bring your child to other clinics or hospitals that are convenient for you.

**Risks and benefits to being in the program:**

By being in the program, you will have access to experienced doctors in the Well Baby Clinic at Hung Vuong hospital who will examine your child for no cost at scheduled visits that will monitor your child’s health and development. The amount of blood we collect from your infant at each visit is small (20-30 drops). Your child will probably cry out when the sample is collected. You will receive money to compensate you for travel costs in bringing your child to the Well Baby Clinic, HVH for follow-up visits in addition to free diagnostic services and supported travel costs for diarrhoeal examinations at HTD. If you bring your child to a private clinic or an emergency department of the hospital other than HTD when your child is sick with diarrhoea, compensation will not be given.

**What we will do with the blood and other samples we collect from your child**

The blood and stool samples we collect from your child will be used for research. The specimens will be stored in a freezer. We will do various tests on the blood, stool and respiratory samples we collect from your child to understand if your child has ever had *Shigella* or other diarrhoeal and respiratory diseases that are common in Vietnam. The tests we do on the blood and respiratory samples we collect from your child will not have a direct benefit to you, but might help other children in the future. Samples we collect from your child may be used in future studies.

**Confidentiality**

All of the information we get from you is strictly private. Your child’s name will not be on any samples we collect or test results–we will use a number instead of a name. Your name, or your child’s name, will not be mentioned in any output from this program. We will ask for information on the district and ward in which you live, but we will not use this information for any purpose outside this program or give it to anyone else.

**Costs**

There will be no cost to you for participating in this program. The costs of examination by a paediatrician at scheduled visits will be paid for by the program. You will be compensated for the travel costs you incur by bringing your child to the clinic for scheduled visits.

**Voluntary participation**

If you do not want to be part of this program, it will not affect the care your child will receive in any way. If you do agree to become a program participant, you can withdraw from the program at any time (verbally). The care your child receives will not be affected. However, information collected on your child up until you withdraw will still be used.

**End of the program**

We will stop seeing your child at HVH after four follow up visits during two years after today. . No information on your child health and no samples will be collected after this time.

**Obtaining additional information**

- You are encouraged to ask any questions related to this program during the time of participation. If you have any questions about this program, its procedures, risks and benefits, or alternatives please call Dr. Nguyễn Trọng Hiếu at 0903 159 285.
- If you have any questions about your child’s rights as a subject in this program, you may want to talk to Dr. Lu Lan Vi, or if you want to speak to someone outside of the program you may contact the Ethics Committee at the HTD at 083 855 8532.

**Consent form for the “Diarrhoeal Cohort Study’ OXTREC – 1058-13**

**Consent from: MOTHER/FATHER/GUARDIAN**

- I have been fully informed of the possible risks and benefits of taking part in this diarrhoeal cohort program and agree that I and my child will take part.
- I know who to contact if I need more information. I understand that confidentiality will be preserved. I understand that I am free to withdraw from the program at any time without affecting the care I or my child will receive.
- I understand there will be limited direct benefit to me or my child.
- I agree to allow nurses to come to my home when I report a diarrhoeal disease episode in my child
- I agree that the geographical location of my house may be recorded in order to understand any relationship between location and disease.

**□ I AGREE OR □ I DO NOT AGREE** that the samples taken can be stored for other research studies about infectious diseases, including genetic testing and these tests may be done outside of Viet Nam.

Participant Number : BCB - ___ - ___ ___ ___ ___

**Participant’s name: ____________________________________________**

**_____________________________________________ _____________________________________________**

**Signature of person giving consent Relationship to participant**

**______________________________________________ ____________________________________________
Print name Date of signature**

**Investigator’s statement**

I, the undersigned, have fully explained the relevant information of this research to the participant named above and will provide her/him with a copy of this signed and dated informed consent form.

____________________________________ ____________________________________ _________________________

**Investigator / designee signature Print name Date of signature**

**If the person giving consent cannot read the form, a witness must be present and sign here:**

I was present throughout the entire informed consent process with the participant. All questions from the participant were answered and the participant has agreed to take part in the research.

____________________________________ ____________________________________ _________________________

**Witness signature Print name Date of signature**

**Consent for genetic testing OXTREC – 1058-13**

**(to be signed by the participant’s parent or guardian)**

By signing/marking my name here, I confirm my willingness for my child’s blood to be used for genetic tests.

Participant Number: BCB - ___ - ___ ___ ___ ___

**Participant’s name: ____________________________________________**

**_____________________________________________ _____________________________________________**

**Signature of person giving consent Relationship to participant**

**______________________________________________ ____________________________________________
Print name Date of signature**

I, the undersigned, have fully explained the relevant information of this research to the participant named above and will provide her/him with a copy of this signed and dated informed consent form.

____________________________________ ____________________________________ _________________________

Investigator / designee signature Print name Date of signature

**If the person giving consent cannot read the form her/himself, a witness must be present and sign here:**

I was present throughout the entire informed consent process with the participant. All questions from the participant were answered and the participant has agreed to take part in the research.

____________________________________ ____________________________________ _________________________

Witness signature Print name Date of signature

- 1. **Vietnamese version for the potential participants coming from the orginal cohort**

| 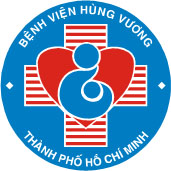 | **Bệnh viện Bệnh Nhiệt Đới**  Đơn vị Nghiên cứu Lâm sàng Đại học Oxford  764 Võ Văn Kiệt, Quận 5, Thành phố Hồ Chí Minh  **Việt Nam** | 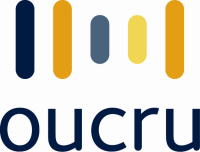 |
| --- | --- | --- |

Bs. Nguyễn Trọng Hiếu

0903159285

**Phiếu thông tin cho “Nghiên cứu Đoàn hệ Bệnh Tiêu chảy”**

**Cho trẻ đã tham gia vào chương trình "Sức khỏe trẻ em"**

**OXTREC - 1058-13**

**Thông tin về chương trình**

Chúng tôi mời những bà mẹ và em bé đã tham gia trong chương trình "Sức Khỏe Trẻ Em" của chúng tôi tại Bệnh viện Hùng Vương cùng tham gia vào **"Nghiên Cứu Đoàn Hệ Bệnh Tiêu Chảy"**. Phiếu này sẽ cung cấp cho bạn những thông tin cần thiết để bạn quyết định có tham gia hay không. Vui lòng đọc kỹ tờ thông tin này. Bạn có thể hỏi về chương trình này, những lợi ích và nguy cơ có thể xảy ra, quyền của người tham gia, và bất kỳ vấn đề nào có liên quan hay thông tin trên phiếu này mà bạn thấy chưa rõ. Khi mọi thắc mắc đã được giải đáp, bạn có thể quyết định cho bạn và con bạn có tham gia hay không. Quá trình này được gọi là sự lấy đồng thuận tham gia nghiên cứu.

Mục tiêu của chương trình này là để xác định tần suất bị bệnh tiêu chảy ở trẻ em Việt Nam, nguyên nhân gây tiêu chảy và tại sao trẻ em lại bị tiêu chảy. Ngoài ra chúng tôi cũng muốn tìm hiểu về việc máu của con bạn phản ứng với bệnh tiêu chảy như thế nào. Chúng tôi hy vọng có thể hiểu hơn về bệnh tiêu chảy ở thành phố Hồ Chí Minh để có thể tạo vắc xin giúp các trẻ em ngừa bệnh sau này.

Đây là một phần của chương trình “Sức khỏe trẻ em” mà bạn đang tham gia. Mục tiêu cụ thể của phần mới này là để tìm hiểu một chủng vi khuẩn có tên gọi *Shigella*, thường gây bệnh tiêu chảy nặng ở trẻ nhỏ. Chúng tôi cần khảo sát thu thập thông tin về nơi ở và điều kiện sống của trẻ em để hiểu hơn về bệnh do vi khuẩn Shigella và các nguyên nhân gây bệnh tiêu chảy khác ở trẻ em Việt Nam. Để thực hiện được điều này, chúng tôi sẽ theo dõi sức khỏe của con bạn trong 2 năm. Trong suốt thời gian đó chúng tôi sẽ theo dõi máu của bé để tìm chứng cứ của nhiễm khuẩn, và sẽ hỏi bạn về tất cả những bệnh mà bé và các thành viên gia đình bạn mắc phải, kể cả những lần phải nhập viện. Những thông tin có được từ các khảo sát này có thể giúp chúng tôi trong việc tạo ra vắc xin ngăn ngừa bệnh tiêu chảy do nhiễm khuẩn *Shigella* trong tương lai.

Chương trình này cũng sẽ có một phần thực hiện xét nghiệm di truyền trên mẫu máu của con bạn. Xét nghiệm di truyền này giúp chúng tôi hiểu vì sao một số người bị bệnh này trong khi một số khác thì không bị. Nếu bạn đồng ý, thông tin di truyền trong máu của con bạn sẽ được xét nghiệm. Kết quả xét nghiệm này sẽ được sử dụng để tiến hành các nghiên cứu, tuy nhiên danh tính của bạn và con bạn sẽ được ẩn để không ai biết thông tin di truyền đó là của ai. Một số mẫu sẽ được gửi ra nước ngoài để xét nghiệm, có thể điều này được cho là xâm phạm đến sự riêng tư. Việc đồng ý cho máu để xét nghiệm di truyền là tùy ở bạn. Bạn và con bạn vẫn có thể tham gia vào chương trình này dù bạn không đồng ý để mẫu máu được dùng cho xét nghiệm di truyền.

**Các đơn vị tham giachương trình?**

Các đơn vị cộng tác tham gia nghiên cứu bao gồm Bệnh viện Hùng Vương (TPHCM) và Bệnh viện Bệnh Nhiệt Đới (TPHCM).

Nếu như tôi thay mặt con tôi chấp thuận tham gia, điều gì sẽ xảy ra trong chương trình và có những nguy cơ thế nào?

**Khi tham gia (hôm nay):**

Khi bé được 12 tháng tuổi và bạn đưa bé đến hẹn khám theo dõi lần cuối cùng cho chương trình Sức Khỏe Trẻ Em ở Phòng khám nhi - Bệnh viện Hùng Vương, bạn và con bạn sẽ được mời tham gia vào "Nghiên cứu Đoàn hệ Bệnh Tiêu Chảy". Nếu bạn và con bạn đồng ý tham gia, một điều dưỡng sẽ cập nhập những thông tin về bạn như nơi bạn ở, cấu trúc gia đình, và tình trạng sức khỏe của bé. Điều dưỡng cũng sẽ lấy 2ml mẫu máu (khoảng 20 giọt) của bé, cho chương trình "Sức khỏe trẻ em" thay vì 1ml (khoảng 10 giọt) như trước đây. Chỉ lấy tối đa 2ml máu từ bé trong lần khám này. Điều dưỡng cũng sẽ lấy một mẫu phân, hoặc một mẫu phết hậu môn nếu không có phân, và mẫu phết mũi họng của con bạn.

**Các lần tái khám (mỗi 6 tháng một lần trong 2 năm):**

Để theo dõi sức khỏe và sự phát triển của con bạn, chúng tôi cần gặp bạn và bé vào mỗi 6 tháng một lần, vào các thời điểm bé được 18, 24, 30 và 36 tháng tuổi.

Mỗi lần tái khám mất khoảng nửa tiếng tại ngay phòng khám nơi bạn được nhận vào nghiên cứu. Điều dưỡng sẽ phát cho bạn một thẻ ghi lịch tái khám của chương trình. Tại mỗi lần tái khám, con của bạn sẽ được bác sĩ kiểm tra sức khỏe. Chúng tôi sẽ ghi lại những thông tin về sự phát triển của con bạn và hỏi thêm một số câu hỏi đơn giản về sức khỏe của bé. Khi con bạn được 18, 24 và 30 tháng, chúng tôi sẽ lấy khoảng 2ml máu (khoảng 20 giọt) của con bạn cho nghiên cứu. Khi con bạn được 36 tháng, chúng tôi sẽ lấy 3ml máu (khoảng 30 giọt). Mỗi lần khám chỉ lấy 1 mẫu máu của bé. Một điều dưỡng nhiều kinh nghiệm sẽ dùng cây kim nhỏ để lấy máu trên tay của bé. Việc lấy máu sẽ gây đau một tí và có thể lại một vết bầm nhỏ. Tại mỗi lần tái khám, điều dưỡng cũng sẽ lấy một mẫu phân hoặc phết hậu môn và một mẫu phết mũi họng của bé. Việc thu mẫu này rất nhanh và không gây đau.

**Khi bạn ở nhà với bé và bạn nghĩ rằng bé bị bệnh tiêu chảy;**

Chúng tôi sẽ giữ liên lạc với bạn bằng tin nhắn SMS định kỳ để hỏi thăm bé có bị tiêu chảy không. Nếu bạn trả lời "Có", một điều dưỡng của chúng tôi sẽ gọi điện cho bạn để sắp xếp thời gian thuận tiện để đến thăm nhà bạn để lấy một mẫu phân, hỏi một số câu hỏi đơn giản và tư vấn y tế để giúp con bạn. Nếu bạn cảm thấy rằng con bạn cần nhập viện, chúng tôi mong bạn xem xét đưa bé đến Bệnh viện Bệnh Nhiệt Đới. Nếu bạn muốn đưa bé đến Bệnh viện Bệnh Nhiệt Đới khi bé bị tiêu chảy, hãy gọi cho điều dưỡng của chúng tôi để được hỗ trợ và bé sẽ được khám bởi một bác sĩ chuyên khoacủa bệnh viện Bệnh Nhiệt Đới . Chúng tôi sẽ thu thập một mẫu phân của bé khi bạn cho bé đến khám tại đây. Bạn cũng có thể đưa bé đến các phòng khám hoặc bệnh viện khác nếu điều đó thuận tiện cho bạn.

**Các nguy cơ và lợi ích khi tham gia:**

Khi tham giachương trình, con bạn sẽ được những bác sĩ có kinh nghiệm tại phòng khám Nhi, bệnh viện Hùng Vương khám và tư vấn miễn phí vào những lần tái khám định kỳ để theo dõi tình trạng sức khỏe và sự phát triển của bé. Lượng máu chúng tôi lấy từ con bạn tại mỗi lần tái khám là rất nhỏ (khoảng 20-30 giọt). Bé có thể khóc khi bị lấy máu. Bạn sẽ nhận được một khoản tiền hỗ trợ cho chi phí đi lại cho các lần tái khám tại phòng khám Nhi, Bệnh viện Hùng Vương, các lần đi khám bệnh vì tiêu chảy tại bệnh viện bệnh Nhiệt đới cũng như miễn phí xét nghiệm khi đến khám tại bệnh viện này vì tiêu chảy. Nếu bạn cho con đến khám ở một phòng khám tư hoặc đến khoa cấp cứu tại một bệnh viện khác, không phải là Bệnh viện Bệnh Nhiệt đới khi bé bị tiêu chảy, chúng tôi sẽ không hỗ trợ các chi phí này.

**Chúng tôi sẽ làm gì với máu và các mẫu khác thu từ con bạn**

Mẫu máu và phân mà chúng tôi thu được từ con của bạn sẽ được dùng vào nghiên cứu. Các mẫu đó sẽ được lưu trữ trong tủ đông lạnh. Chúng tôi sẽ làm một số xét nghiệm trên mẫu máu, phân và mẫu phết mũi họng của con bạn để xem con bạn có từng bị nhiễm *Shigella* hoặc một số bệnh tiêu chảy và hô hấp phổ biến khác ở Việt Nam hay không. Những xét nghiệm mà chúng tôi tiến hành trên các mẫu máu và mũi họng thu được từ con bạn có thể không mang lại lợi ích trực tiếp cho bạn, nhưng điều này có thể giúp những trẻ em khác trong tương lai. Các mẫu chúng tôi thu được từ con bạn cũng có thể được sử dụng cho các nghiên cứu về sau.

**Tính bảo mật**

Tất cả những thông tin chúng tôi có được từ bạn sẽ được bảo mật nghiêm ngặt. Tên của con bạn sẽ không hiện diện trên bất kì một mẫu xét nghiệm nào cũng như trên kết quả của chúng tôi - chúng tôi sẽ dùng mã số thay cho tên. Tên của bạn, tên của con bạn sẽ không được đề cập đến trên kết quả của chương trình. Chúng tôi sẽ hỏi thông tin về quận và phường nơi bạn ở nhưng sẽ không sử dụng thông tin này vào bất kỳ mục đích nào khác ngoài chương trình này và cũng không đưa cho ai khác.

**Chi phí**

Bạn không tốn bất kì chi phí nào khi tham gia vào chương trình này. Việc khám bệnh theo lịch sẽ được chương trình chi trả. Bạn sẽ được hỗ trợ chi phí đi lại khi mang bé đến phòng khám để tái khám theo lịchđã hẹn. Bạn cũng được miễn phí chi phí xét nghiệm chẩn đoán bệnh và được hỗ trợ chi phí đi lại khi cho bé đến khám tại bệnh viện Bệnh Nhiệt Đới vì tiêu chảy.

**Sự tự nguyện tham gia chương trình**

Dù bạn chọn không tham gia vào chương trình thì việc đó cũng không ảnh hưởng đến việc chăm sóc sức khỏe cho con bạn. Ngay cả khi bạn đã đồng ý tham gia chương trình, bạn cũng có thể xin rút khỏi chương trình bất kỳ lúc nào (bằng lời). Việc chăm sóc sức khỏe cho con bạn sẽ không bị ảnh hưởng. Tuy nhiên những thông tin đã được thu thập về con bạn cho đến thời điểm đó vẫn sẽ được dùng cho nghiên cứu.

**Kết thúc chương trình**

Chúng tôi sẽ ngừng khám theo dõi con bạn sau bốn lần theo dõi trong vòng 2 năm tại bệnh viện Hùng Vương kể từ ngày hôm nay. Sau đó, chúng tôi sẽ không thu thập thêm mẫu và thông tin từ con bạn nữa.

**Các thông tin thêm**

- Chúng tôi khuyến khích bạn hỏi thêm bất kỳ câu hỏi gì liên quan đến chương trình này trong suốt thời gian tham gia. Nếu bạn có thắc mắc về chương trình, các quy trình, nguy cơ và lợi ích, hay các câu hỏi khác, vui lòng gọi BS. Nguyễn Trọng Hiếu tại số 0903 159 285
- Nếu bạn có bất kỳ thắc mắc gì về các quyền của con bạn khi tham gia chương trình này, bạn có thể liên hệ BS. Lư Lan Vi hoặc nếu bạn muốn nói chuyện với ai khác ngoài nhóm nghiên cứu, bạn có thể liên hệ với Hội Đồng Đạo Đức tại Bệnh viện Bệnh Nhiệt đới ở số 083 855 8532.

**Phiếu chấp thuận tham gia "Nghiên cứu Đoàn hệ Bệnh Tiêu chảy" OXTREC – 1058-13**

**Chấp thuận từ: BỐ/MẸ/NGƯỜI GIÁM HỘ**

- Tôi đã được thông tin đầy đủ về các nguy cơ và lợi ích có thể có của việc cho con tôi tham gia vào chương trình nghiên cứu đoàn hệ bệnh tiêu chảy này và đồng ý rằng tôi và con của tôi sẽ tham gia.
- Tôi biết sẽ liên lạc với ai khi cần hỏi thêm thông tin. Tôi hiểu rằng các thông tin về tôi hay con tôi sẽ được bảo mật. Tôi hiểu được tôi có quyền rút khỏi chương trình vào bất kỳ lúc nào mà không ảnh hưởng đến việc chăm sóc sức khỏe mà tôi hay con tôi sẽ được nhận.
- Tôi hiểu là việc nghiên cứu này sẽ không mang đến nhiều lợi ích trực tiếp cho tôi và con tôi
- Tôi đồng ý để điều dưỡng của chương trình đến nhà tôi khi tôi báo con tôi bị nhiễm bệnh tiêu chảy
- Tôi đồng ý việc định vị nhà ở của tôi có thể được thực hiện để tìm hiểu sự liên quan giữa nơi ở và bệnh tật.

**□ Tôi đồng ý** hoặc **□ Tôi không đồng ý** cho phép mẫu của con tôi được lưu trữ cho các nghiên cứu về sau về các bệnh truyền nhiễm, bao gồm các xét nghiệm di truyền và các xét nghiệm này có thể được thực hiện bên ngoài Việt Nam.

Mã số tham gia: 14EN - ___ - ___ ___ ___ ___

**Tên người tham gia: ____________________________________________**

**______________________________________ ______________________________________**

**Chữ ký của người chấp thuận Quan hệ với người tham gia**

**______________________________________ ______________________________________**

**Họ Tên (chữ in) Ngày ký**

**Xác nhận của nghiên cứu viên**

Tôi, người ký tên bên dưới, đã giải thích đầy đủ các thông tin liên quan đến chương trình nghiên cứu này cho người tham gia có tên bên trên và sẽ cung cấp cho cô/anh ấy một bản sao của phiếu chấp thuận đã được ký và ghi ngày tháng này.

**______________________________ _____________________________** **____________________________** **Chữ ký của nghiên cứu viên Họ Tên (chữ in) Ngày ký**

**hoặc người được chỉ định**

**Nếu người chấp thuận không thể tự đọc phiếu này, một nhân chứng phải có mặt và ký tên dưới đây:**

Tôi đã có mặt cùng với người tham gia trong suốt quá trình lấy chấp thuận. Tất cả các câu hỏi của người tham gia đã được trả lời và người tham gia đã đồng ý tham gia vào chương trình nghiên cứu.

**______________________________ _______________________________ ________________________**

**Chữ ký nhân chứng Họ Tên (chữ in) Ngày ký**

**Phiếu chấp thuận cho xét nghiệm di truyền OXTREC - 1058-13**

**(được ký tên bởi bố mẹ hoặc người giám hộ của trẻ tham gia nghiên cứu)**

Bằng việc ký tên/đánh dấu ở đây, tôi xác nhận đồng ý cho phép mẫu máu của con tôi được dùng vào các xét nghiệm di truyền.

Mã số tham gia: 14EN-___-___ ___ ___ ___

**Tên người tham gia: ____________________________________________**

**______________________________________ ______________________________________**

**Chữ ký của người chấp thuận Quan hệ với người tham gia**

**______________________________________ ______________________________________**

**Họ Tên (chữ in) Ngày ký**

Tôi, người ký tên bên dưới, đã giải thích đầy đủ các thông tin liên quan đến chương trình nghiên cứu này cho người tham gia có tên bên trên và sẽ cung cấp cho cô/anh ấy một bản sao của phiếu chấp thuận đã được ký và ghi ngày tháng này.

**______________________________ _____________________________** **____________________________** **Chữ ký của nghiên cứu viên Họ Tên (chữ in) Ngày ký**

**hoặc người được chỉ định**

**Nếu người cho chấp thuận không thể tự đọc phiếu này, một nhân chứng phải có mặt và ký tên dưới đây:**

Tôi đã có mặt cùng với người tham gia trong suốt quá trình lấy chấp thuận. Tất cả các câu hỏi của người tham gia đã được trả lời và người tham gia đã đồng ý tham gia vào chương trình nghiên cứu.

**______________________________ _______________________________ ________________________**

**Chữ ký nhân chứng Họ Tên (chữ in) Ngày ký**

1. **ICFs for the community survey**

**2.1 English version for the community survey**

**PARTICIPANT INFORMATION SHEET AND INFORMED CONSENT FORM**

**Diarrhoea Management among children under 5 in the community in Ho Chi Minh City, Vietnam**

**Introduction and summary**

You are invited to take part in this research study to help us explore behavior for the treatment of diarrhoeal disease among children less than five years of age in the community. We want to know whichoptions for outside-the-home healthcare you would seek if your child had an episode of diarrhoeal disease. We also want to know about your attitude on the use of antimicrobials and antimicrobial resistance issues.

You will have a copy of this sheet to keep for your records.

**What do I do if I participate in the study?**

We will ask you to sign in the inform consent form and we will ask you a few questions regarding your demographics, your child’s health status during the last month, what you would do if your child has diarrhoea and where would you choose to seek healthcare outside of the home. We also ask you some questions on your attitude regarding antimicrobial usage and antimicrobial resistance. The interview will take approximately 15 minutes.

You are free to no answer any questions that you do not want to answer.

**What happens to the information I give?**

The interview will be private between yourself and the interviewer. The questionnaire is anonymous so no one will know the answers are yours. Only the project team members have access to your answers but your information will be anonymous.

**What are the risks and the benefits if I participate in the study?**

There are no disadvantages if you decide to participate, except that you need to spend time for the interview. Your answers will contribute to the understanding of management of diarrhoea in the community in Vietnam, and help inform future public health promotion for diarrhoeal diseases in Vietnamese children. There are also no disadvantages if you choose not to participate.

You will receive a compensation for the time you spend taking part in this study (50,000VND).

**Is my participation is compulsory or voluntary?**

Your choice to participate is completely voluntary. You are free to decide whether you want to join in this project. If you agree you can then withdraw from the project at any time without any consequences. You can also withdraw any given information before the analysis process.

**When I need some more information, who should I contact?**

We encourage you to discuss any problems with the study team. If you need any more information about the participation, you can contact Ms Le Thi Quynh Nhi at 0946309385

**INFORMED CONSENT FORM**

**Diarrhoea Management In Ho Chi Minh City, Vietnam**

- I have read the information given to me and freely agree to be in this study. I will be given a copy of this form to keep.
- I have been told about the risks and benefits.
- I understand that I may stop participating in the study at any time.

**(Please check one)**

- □ **I AGREE OR** □ **I DO NOT AGREE** to take part in the interview.

**By signing/marking my name here, I confirm what is written above**

| Signature of Participant:  ________________________ | Full Name:  ___________________________ | Date of Signature:  ____/____/_____ |
| --- | --- | --- |

I, the undersigned, have fully explained the relevant information of this study to the person named above and will provide her/him with a copy of this signed and dated informed consent form.

| ___________________ | _______________ | ___/____/_____ |
| --- | --- | --- |
| Investigator/Designee Signature | Print Name | Date of Signature |

**If the person giving consent cannot read the form themselves, a witness must be present and sign here: I was present throughout the entire informed consent process with the participant. This form was read accurately to the volunteer, all questions from the volunteer were answered and the volunteer has agreed to take part in the research.**

| _______________ | ________________________________________ | ___/____/_____ |
| --- | --- | --- |
| Witness Signature | Full Name | Date of Signature |

**2.2 Vietnamese version of the ICF for community survey**

**BẢNG THÔNG TIN VÀ PHIẾU CHẤP THUẬN THAM GIA KHẢO SÁT**

**Khảo sát về thực trạng xử trí tiêu chảy cho trẻ em dưới 5 tuổi trong cộng đồng tại thành phố Hồ Chí Minh, Việt Nam**

**Giới thiệu và Tóm tắt**

Chúng tôi mời bạn tham gia vào khảo sát thực hành của ba mẹ hoặc người chăm sóc trẻ dưới 5 tuổi khi trẻ bị tiêu chảy trong cộng đồng. Chúng tôi muốn biết anh/ chị sẽ lựa chọn những dịch vụ y tế nào khi có tình huống bé bị tiêu chảy. Chúng tôi cũng muốn tìm hiểu thái độ của anh chị trong việc sử dụng thuốc kháng sinh và vấn đề kháng kháng sinh.

Chúng tôi có 02 bản giống hệt nhau và sẽ gửi cho anh chị 01 bản để giữ.

**Tôi sẽ làm gì khi tham gia khảo sát này?**

Nếu anh/ chị đồng ý tham gia, anh chị sẽ ký tên vào “Phiếu chấp thuận tham gia” và chúng tôi sẽ hỏi một vài câu hỏi về các đặc điểm liên quan đến dân số của anh/ chị, vài câu về tình trạng sức khỏe của trẻ trong vòng 01 tháng qua, chúng tôi sẽ hỏi về lựa chọn anh chị sẽ thực hiện nếu trẻ bị tiêu chảy. Sẽ mất khoảng 15 phút cho việc phỏng vấn.

Anh chị được quyền trả lời hoặc từ chối trả lời bất cứ câu hỏi nào anh chị không muốn.

**Những thông tin tôi cung cấp sẽ được dùng làm gì?**

Nghiên cứu này sử dụng bộ câu hỏi ẩn danh nên không ai được biết anh/ chị đã trả lời phiếu câu hỏi nào. Chỉ có nhóm nghiên cứu mới được tiếp cận các thông tin anh/ chị đã trả lời nhưng trên đó không ghi rõ họ và tên anh/ chị.

**Các nguy cơ và bất lợi nếu tôi tham gia?**

Đây là khảo sát thông qua phỏng vấn hỏi – trả lời nên nếu anh chị đồng ý tham gia, sẽ không có bất lợi gì về sức khỏe hoặc bất cứ bất lợi gì khác, ngoại trừ mất một chút thời gian trả lời câu hỏi.

Các câu trả lời của anh/ chị sẽ giúp chúng tôi hiểu được cách xử trí của người dân trong cộng đồng nói chung khi trẻ bị tiêu chảy, từ đó sẽ giúp cho việc thông tin đến các chiến lược truyển thông nhằm cải thiện tình hình tiêu chảy ở Việt Nam.

Nếu anh/ chị không tham gia thì cũng không có bất lợi nào cho mình.

Anh/ chị sẽ được gửi một món quà nhỏ (trị giá 50.000VND) để cảm ơn thời gian anh/ chị đã dành cho trả lời câu hỏi.

**Tham gia tự nguyện hay bắt buộc?**

Việc tham gia hoàn toàn tự nguyện. Anh/ chị hoàn toàn được tự ý quyết định tham gia hoặc không tham gia. Anh/ chị có thể rút lui ở bất kỳ thời điểm nào mà không bị ảnh hưởng gì cả. Nếu anh/ chị muốn rút khỏi khảo sát, bất cứ lúc nào cũng được, trước giai đoạn các dữ liệu được phân tích.

**Tôi cần biết thêm thông tin, tôi liên hệ với ai?**

Chúng tôi khuyến khích anh/ chị tham gia thảo luận bất cứ vấn đề gì liên quan với nhóm nghiên cứu. Khi cần anh/ chị có thể liên hệ: ThS. Lê Thị Quỳnh Nhi, 0946309385.

**PHIẾU CHẤP THUẬN THAM GIA**

**Khảo sát về thực trạng xử trí tiêu chảy cho trẻ em dưới 5 tuổi trong cộng đồng tại thành phố Hồ Chí Minh, Việt Nam**

- Tôi đã đọc và hiểu thông tin trên đây, đã có cơ hội xem xét và đặt câu hỏi về thông tin liên quan đến nội dung trong nghiên cứu này.
- Tôi đã nói chuyện trực tiếp về nguy cơ và lợi ích
- Tôi sẽ nhận một bản sao của Bản Thông tin và phiếu chấp thuận tham gia.
- Tôi hiểu rằng mình có thể dừng tham gia khảo sát bất cứ lúc nào.

Tôi chọn

□**TÔI ĐỒNG Ý THAM GIA** □ **TÔI KHÔNG ĐỒNG Ý THAM GIA**

**Chữ ký của người tham gia:**

Họ tên người tham gia__________________________________ Chữ ký___________________

Ngày tháng năm____/______/_______

**Chữ ký của người làm chứng hoặc của người đại diện hợp pháp (nếu áp dụng):**

Họ tên người làm chứng________________________________ Chữ ký ___________________

Ngày tháng năm_____/______/______

**Chữ ký của Nghiên cứu viên/người lấy chấp thuận:**

Tôi, người ký tên dưới đây, xác nhận rằng bệnh nhân/người tình nguyện tham gia nghiên cứu ký bản chấp thuận đã đọc toàn bộ bản thông tin trên đây, các thông tin này đã được giải thích cặn kẽ cho Anh/ Chị và Anh/ Chị đã hiểu rõ bản chất, các nguy cơ và lợi ích của việc tham gia vào khảo sát này.

Họ tên nghiên cứu viên ________­­­­­­­­­­­­­­­­_________________________ Chữ ký___________________

Ngày tháng năm____/_______/______
